# Catalytic Enantioselective Cyclopropylalkynylation of Aldimines Generated In Situ from α-Amido Sulfones

**DOI:** 10.3390/molecules27123763

**Published:** 2022-06-11

**Authors:** Alicia Monleón, Gonzalo Blay, José R. Pedro

**Affiliations:** Departament de Química Orgànica, Facultad de Química, Universitat de València, C/Dr. Moliner 50, 46100 Burjassot, Spain; alicia.monleon@uv.es

**Keywords:** *N*-carbamoyl-protected propargylic amines, α-amido sulfones, cyclopropylacetylene, BINOL, zinc complex

## Abstract

A convenient procedure of synthesis of *N*-carbamoyl-protected propargylic amines substituted with a cyclopropyl group from α-amido sulfones and cyclopropylacetylene is described. The reaction is catalyzed by a chiral BINOL-type zinc complex and provides the corresponding products in good yields and enantioselectivities.

## 1. Introduction

Propargylic amines are an important class of alkyne-coupled nitrogen compounds used as building blocks for the synthesis of biologically active compounds of interest in the pharmaceutical and agrochemical industries [1,2,3]. Recently, their application in the chemical fixation of carbon dioxide has been also described [4,5,6]. Consequently, a number of methodologies detailing the preparation of these building blocks have been disclosed, mostly involving the direct addition of alkynyl nucleophiles to imines [7,8,9,10,11,12].

On the other hand, the presence of the cyclopropyl group in organic compounds has attracted enormous attention due to its exceptional steric, electronic, and conformational properties as a consequence of its tensioned structure [13,14,15]. Its presence in nature (terpenes, antibiotics, pheromones, or fatty acids) has led to the search for new biologically active compounds incorporating this ring. In fact, several drugs with antitumor, anti-inflammatory, or antimalarial activity, among others, have been developed [16]. In some cases, such as Efavirenz (Sustiva^®^, Bristol Myers Squibb), the cyclopropylacetylene group is present in its structure [17,18,19,20,21].

**Scheme 1:**
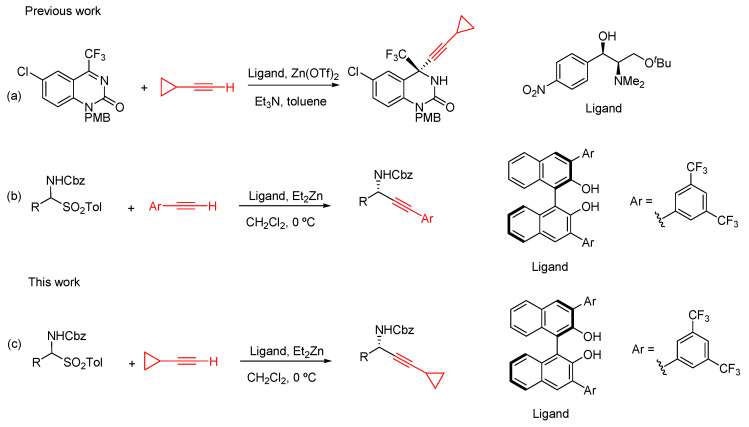
Enantioselective alkynylation of ketimines and α-amido sulfones. (**a**) Enantioselective cyclopropylakynylation of trifluoromethylketimines [22]. (**b**) Enantioselective alkynylation of α-amido sulfones [23]. (**c**) Enantioselective cyclopropylalkynylation of α-amido sulfones.

The catalytic enantioselective addition of cyclopropylacetylene to the C=N bond of imines would allow the cyclopropyl group to be easily introduced as a substituent in a propargylic amine. However, despite extensive studies on the catalytic asymmetric alkynylation of imines, only one example of enantioselective cyclopropylalkynylation reaction using a stoichiometric amount of a chiral ligand has been described by Jiang and Si (Scheme 1a) [22]. In particular, this procedure involves the enantioselective cyclopropylalkynylation of a cyclic *N*-acyl ketimine using 1.1 equivalents of a chloramphenicol-derived chiral ligand and zinc triflate in the presence of 2.5 equivalents of triethylamine. This procedure allowed them to synthesize DPC-961, a second-generation non-nucleoside HIV reverse transcriptase inhibitor analogous to Efavirenz.

In this kind of reaction, the substituent on the nitrogen atom of the imine plays a crucial role, thus strongly affecting the electrophilicity of the imine. Moreover, the synthetic utility of this reaction also depends on the ease of elimination of this substituent in the resulting amines. The *tert*-butoxycarbonyl (BOC) and benzyloxycarbonyl (Cbz) protecting groups fulfill these requirements, but the corresponding *N*Boc and *N*Cbz imines are relatively unstable. However, by using the corresponding α-amido sulfones, it is possible to prepare these imines in situ to avoid their synthesis and isolation, thus circumventing their instability (Scheme 1b) [24,25,26].

In this paper, we describe the enantioselective cyclopropylalkynylation of aldimines generated in situ from α-amido sulfones, catalyzed by a chiral BINOL-type zinc complex, as a convenient procedure of synthesis of *N*-carbamoyl-protected propargylic amines substituted with a cyclopropyl group (Scheme 1c).

## 2. Results and Discussion

Having in mind the importance of α-amido sulfones as a convenient starting material of imine, we carried out the preparation of a set of 15 α-amido sulfones 1 from the corresponding aldehydes, following the method described by Engberts [27].

Starting from amido sulfone **1a**, we conducted the addition of cyclopropylacetylene **2** under the same conditions used by our group in the addition of phenylacetylene and other arylacetylenes to different α-amido sulfones [23]. Thus, a 1 M Et_2_Zn solution in hexanes (3 eq.) was added dropwise to a mixture of chiral BINOL-type ligand **L** (0.2 eq.) and cyclopropylacetylene (7.2 eq.) in CH_2_Cl_2_ at room temperature. After stirring for 2 h, the reaction mixture was cooled to 0 °C and then a solution of the α-amido sulfone **1a** (1 eq.) in CH_2_Cl_2_ was added. Under these reaction conditions the corresponding propargylic Cbz-amide **3a** was obtained with good yield (72%) and enantiomeric excess (86% e.e.)

**Scheme 2:**
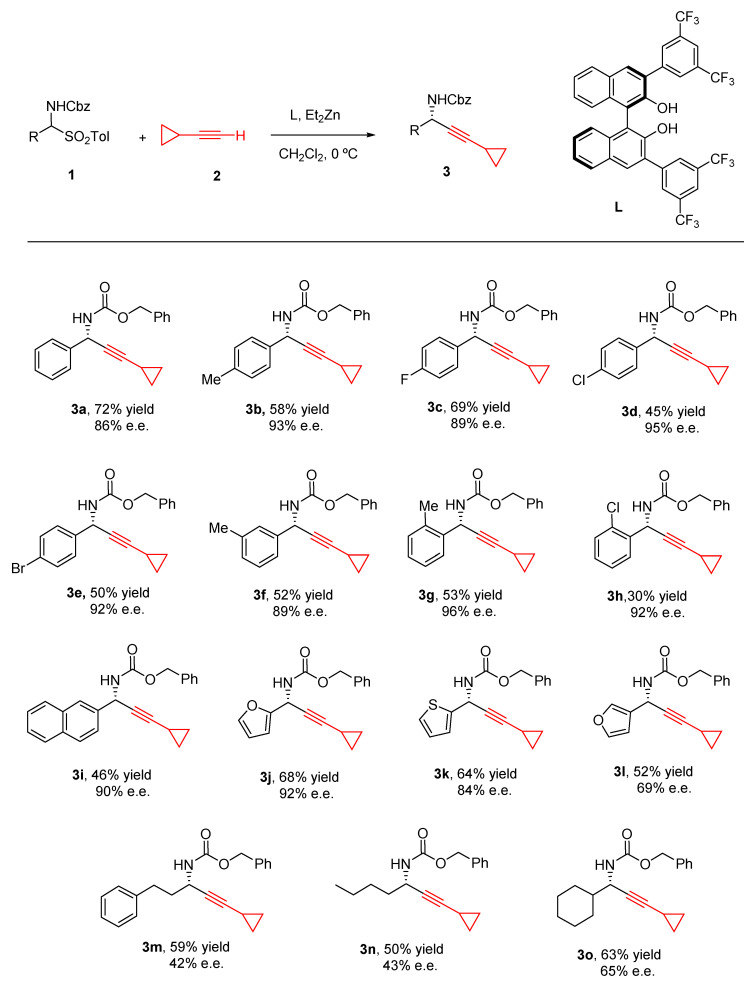
Enantioselective addition of cyclopropylacetylene (**2**) to *N*-benzyloxycarbonyl-*para*-toluenesulfones **1**. Substrate scope study.

These reaction conditions were used for the addition of cyclopropylacetylene **2** to several *N*-benzyloxycarbonyl-*para*-toluenesulfones **1** derived from substituted benzaldehydes with good results (Scheme 2). Both electron-donating (Me) and electron-withdrawing (F, Cl, Br) substituents in *para*, *meta,* and *ortho* positions were well tolerated, providing the expected products with good yields and enantioselectivities ranging from 89 to 96% ee. It is interesting to remark on the results obtained with the α-amido sulfones derived from *ortho*-substituted benzaldehydes. In the case of the *ortho*-methyl derivative, the reaction product was obtained with an excellent 96% ee, whereas in the case of the larger *ortho*-chloro the reaction product was obtained with a 92% e.e., although in this second case the yield was lower (30%). Bulky α-amido sulfone **1i** derived from 2-naphthylcarbaldehyde gave the corresponding product **3i** with a very good ee value (90%) and acceptable yield (46%).

α-Amido sulfones (**1j**–**1l**) derived from heteroaromatic aldehydes bearing electron-rich five-membered heterocycles were also suitable substrates. On the other hand, α-amido sulfones derived from 2-furan (**1j**) and 2-thiophene carbaldehyde (**1k**) give the reaction products with very good enantiomeric excesses (92% and 84%) and yields (68% and 64%); α-amido sulfone derived from 3-furan carbaldehyde (**1l**) gives poorer results (69% e.e. and 52% yield). Finally, the aliphatic α-amido sulfones **1m**–**o** reacted with cyclopropylacetylene **2** to give the corresponding propargylic Cbz-amides **3m**–**o** with moderate yields (50–63%) and enantioselectivities (42–65% e.e.). In these cases, substrates bearing a secondary α-alkyl substituent (**1o**) give better results (63% yield, 65% e.e.) than those derived from linear alkyl aldehydes (**1m**–**n**) (50–59% yield, 42–43% e.e.)

The stereochemistry of the newly formed stereogenic center in the resulting products was assigned to be *S* under the assumption of a uniform mechanistic pathway as that of the reaction of α-amido sulfones with phenylacetylene under the same reaction conditions [23,28].

## 3. Materials and Methods

### 3.1. General Information

All reactions were performed under an inert atmosphere of nitrogen in round-bottomed flasks oven-dried overnight at 120 °C. All commercial reagents were used as received without additional purification. Dichloromethane was distilled from CaH_2_ and stored over molecular sieves (4 Å). Reactions were monitored by thin-layer chromatography (TLC) analysis using silica gel 60 (F-254 plates). Flash column chromatography was performed using silica gel 60 (0.040–0.063 mm). All new compounds were characterized by NMR spectroscopy (^1^H and ^13^C), high-resolution mass spectroscopy (HRMS), and melting point (if solids). NMR spectra were recorded at 300 MHz for ^1^H and at 75 MHz for ^13^C NMR. ^1^H NMR spectra and ^13^C NMR spectra were internally referenced to CHCl_3_ (δ = 7.26 and 77.0 ppm, respectively). Chemical shifts are reported in ppm. The carbon type was determined by DEPT experiments. The following abbreviations are used to indicate the multiplicity in NMR spectra: s, singlet; d, doublet; t, triplet; q, quartet; m, multiplet; br.s, broad signal. The HRMS experiments were performed with a Q-Tof Premier Mass Spectrometer, equipped with an electrospray ionization (ESI) source. The drying gas and also the nebulizing gas was nitrogen. Specific optical rotations were measured with the use of sodium light (D line 589 nm). Chiral HPLC analyses were performed with a chromatograph and a UV diode array detector on chiral stationary phase columns. The abbreviation Rt is used to indicate the retention time of an enantiomer. α-Amido sulfones **1** were prepared from the corresponding aldehyde, carbamate, and sodium *para*-toluenesulfinate, as described in the literature [27].

### 3.2. General Procedure for the Enantioselective Cyclopropylalkynylation of Compounds ***1***

A 1M solution of Et_2_Zn in hexane (0.375 mL, 0.375 mmol) was added dropwise at room temperature under nitrogen to a solution of ligand L (17.7 mg, 0.025 mmol) and cyclopropylacetylene **2** (0.9 mmol) in CH_2_Cl_2_ (0.4 mL). After stirring for 1.5 h, the reaction mixture was cooled to 0 °C. After 15 min, a solution of α-amido sulfone **1** (0.125 mmol) in CH_2_Cl_2_ (1.0 mL) was added by syringe. The solution was stirred until the reaction was complete (TLC). The reaction mixture was quenched with water (1.0 mL), extracted with CH_2_Cl_2_ (3 × 15 mL), dried over MgSO4, and concentrated under reduced pressure. Purification by flash chromatography on silica gel afforded compound **3**. (^1^H-NMR and ^13^C-NMR and HPLC traces of compounds **3a**–**o** are shown in Supplementary Materials.)

Benzyl (*S*)-(3-cyclopropyl-1-phenylprop-2-yn-1-yl)carbamate (**3a**)

Compound **3a** was obtained as a white solid with a yield of 72%; m.p. 102–104 °C; [α]^D^_20_ −3.1 (c 1.01, CHCl_3_, 86% ee); enantiomeric excess (86%) was determined by chiral HPLC (Chiralcel OD-H), hexane/*^i^*PrOH 90:10, 1 mL/min, major enantiomer Rt = 13.4 min, minor enantiomer Rt = 9.5 min; ^1^H NMR (300 MHz, CDCl_3_) δ 7.48 (d, *J* = 7.1 Hz, 2H), 7.38–7.29 (m, 8H), 5.66 (d, *J* = 7.9 Hz, 1H), 5.22 (d, *J* = 6.5 Hz, 1H), 5.16 (d, *J* = 12.1 Hz, 1H), 5.10 (d, *J* = 12.4 Hz, 1H), 1.34 (m, 1H), 0.82–0.76 (m, 2H), 0.74–0.68 (m, 2H); ^13^C NMR (75.5 MHz, CDCl_3_) δ 155.3 (C), 139.6 (C), 136.2 (C), 128.6 (CH), 128.5 (CH), 128.1 (CH), 127.9 (CH), 126.9 (CH), 88.8 (C), 73.1 (C), 67.0 (CH_2_), 47.1 (CH), 8.2 (CH_2_), −0.5 (CH); HRMS (ESI) *m*/*z*: [M + H]^+^, calcd for C_20_H_20_NO_2_ 306.1489, found 306.1489.

Benzyl (*S*)-(3-cyclopropyl-1-(4-tolyl)prop-2-yn-1-yl)carbamate (**3b**)

Compound **3b** was obtained as a white solid with a yield of 58%; m.p. 104–107 °C; [α]^D^_20_ −9.4 (c 1.00, CHCl_3_, 93% ee); enantiomeric excess (93%) was determined by chiral HPLC (Chiralcel AD-H), hexane/*^i^*PrOH 90:10, 1 mL/min, major enantiomer Rt = 11.9 min, minor enantiomer Rt = 12.6 min; ^1^H NMR (300 MHz, CDCl_3_) δ 7.37–7.29 (m, 7H), 7.15 (d, *J* = 8.0 Hz, 2H), 5.19–5.13 (m, 2H), 5.09 (d, *J* = 12.4 Hz, 1H), 2.34 (s, 3H), 1.33–1.24 (m, 1H), 0.81–0.75 (m, 2H), 0.73–0.67 (m, 2H); ^13^C NMR (75.5 MHz, CDCl_3_) δ 155.3 (C), 137.7 (C), 136.8 (C), 136.3 (C), 129.2 (CH), 128.5 (CH), 128.1 (CH), 126.8 (CH), 88.6 (C), 73.3 (C), 67.0 (CH_2_), 46.8 (CH), 21.1 (CH_3_), 8.2 (CH_2_), −0.5 (CH); HRMS (ESI) *m*/*z*: [M + H]^+^, calcd for C_21_H_22_NO_2_ 320.1645, found 320.1643.

Benzyl (*S*)-(3-cyclopropyl-1-(4-fluorophenyl)prop-2-yn-1-yl)carbamate (**3c**)

Compound **3c** was obtained as a white solid with a yield of 69%; m.p. 116–117 °C; [α]^D^_20_ −16.6 (c 1.02, CHCl_3_, 89% ee); enantiomeric excess (89%) was determined by chiral HPLC (Chiralcel AD-H), hexane/*^i^*PrOH 90:10, 1 mL/min, major enantiomer Rt = 12.8 min, minor enantiomer Rt = 11.5 min; ^1^H NMR (400 MHz, CDCl_3_) δ 7.47–7.42 (m, 2H), 7.35–7.31 (m, 5H), 7.02 (t, *J* = 8.7 Hz, 2H), 5.63 (d, *J* = 7.7 Hz, 1H), 5.22 (d, *J* = 5.4 Hz, 1H), 5.15 (d, *J* = 12.1 Hz, 1H), 5.09 (d, *J* = 12.1 Hz, 1H), 1.33–1.24 (m, 1H), 0.82–0.74 (m, 2H), 0.73–0.67 (m, 2H); ^13^C NMR (100 MHz, CDCl_3_) δ 162.4 (d, J = 246.5 Hz, C), 155.3 (C), 136.2 (C), 135.6 (d, *J* = 3.2 Hz, C), 128.7 (d, *J* = 8.3 Hz, CH), 128.5 (CH), 128.2 (CH), 128.1 (CH), 115.4 (d, *J* = 21.6 Hz, CH), 89.1 (C), 72.6 (C), 67.1 (CH_2_), 46.5 (CH), 8.2 (CH_2_), −0.6 (CH); ^19^F NMR (282 MHz, CDCl_3_) δ –114.9; HRMS (ESI) *m*/*z*: [M + H]^+^, calcd for C_20_H_19_NO_2_F 324.1394, found 324.1389.

Benzyl (*S*)-(1-(4-chlorophenyl)-3-cyclopropylprop-2-yn-1-yl)carbamate (**3d**)

Compound **3d** was obtained as a pale yellow solid with a yield of 45%; m.p. 116–117 °C; [α]^D^_20_ −6.3 (c 1.05, CHCl_3_, 95% ee); enantiomeric excess (95%) was determined by chiral HPLC (Chiralcel AD-H), hexane/*^i^*PrOH 90:10, 1 mL/min, major enantiomer Rt = 12.5 min, minor enantiomer Rt = 12.0 min; ^1^H NMR (300 MHz, CDCl_3_) δ 7.42–7.29 (m, 9H), 5.61 (d, *J* = 7.9 Hz, 1H), 5.20 (d, *J* = 6.5 Hz, 1H), 5.15 (d, *J* = 12.1 Hz, 1H), 5.09 (d, *J* = 12.8 Hz, 1H), 1.33–1.23 (m, 1H), 0.32–0.74 (m, 2H), 0.72–0.67 (m, 2H); ^13^C NMR (75.5 MHz, CDCl_3_) δ 155.3 (C), 138.3 (C), 136.1 (C), 133.7 (C), 128.7 (CH), 128.5 (CH), 128.3 (CH), 128.2 (CH), 128.1 (CH), 89.3 (C), 72.3 (C), 67.2 (CH_2_), 46.5 (CH), 8.2 (CH_2_), −0.6 (CH); HRMS (ESI) *m*/*z*: [M + H]^+^, calcd for C_20_H_19_NO_2_Cl 340.1099, found 340.1097.

Benzyl (*S*)-(1-(4-bromophenyl)-3-cyclopropylprop-2-yn-1-yl)carbamate (**3e**)

Compound **3e** was obtained as a white solid with a yield of 50%; m.p. 131–133 °C; [α]^D^_20_ −3.6 (c 1.06, CHCl_3_, 92% ee); enantiomeric excess (92%) was determined by chiral HPLC (Chiralcel IC-H), hexane/*^i^*PrOH 98:2, 1 mL/min, major enantiomer Rt = 22.2 min, minor enantiomer Rt = 23.8 min; ^1^H NMR (300 MHz, CDCl_3_) δ 7.46 (d, *J* = 8.5 Hz, 2H), 7.39–7.30 (m, 7H), 5.59 (d, *J* = 8.3 Hz, 1H) 5.20–5.13 (m, 2H), 5.08 (d, *J* = 12.1 Hz, 1H), 1.32–1.23 (m, 1H), 0.82–0.74 (m, 2H), 0.72–0.66 (m, 2H). ^13^C NMR (75.5 MHz, CDCl_3_) δ 155.2 (C), 138.8 (C), 136.1 (C), 131.6 (CH), 128.6 (CH), 128.5 (CH), 128.2 (CH), 128.1 (CH), 121.9 (C), 89.3 (C), 72.5 (C), 67.2 (CH_2_), 46.6 (CH), 8.2 (CH_2_), −0.6 (CH); HRMS (ESI) *m*/*z*: [M + H]^+^, calcd for C_20_H_19_NO_2_Br 384.0594, found 384.0591.

Benzyl (*S*)-(3-cyclopropyl-1-(3-tolyl)prop-2-yn-1-yl)carbamate (**3f**)

Compound **3f** was obtained as a white solid with a yield of 52%; m.p. 81–83 °C; [α]^D^_20_ −10.6 (c 1.01, CHCl_3_, 89% ee); enantiomeric excess (89%) was determined by chiral HPLC (Chiralcel OD-H), hexane/*^i^*PrOH 90:10, 1 mL/min, major enantiomer Rt = 11.1 min, minor enantiomer Rt = 9.3 min; ^1^H NMR (300 MHz, CDCl_3_) δ 7.36–7.31 (m, 5H), 7.26–7.21 (m, 3H), 7.11 (d, *J* = 7.0 Hz, 1H), 5.62 (d, *J* = 7.8 Hz, 1H), 5.21–5.14 (m, 2H), 5.10 (d, *J* = 12.4 Hz, 1H), 2.35 (s, 3H), 1.34–1.24 (m, 1H), 0.82–0.75 (2H), 0.74–0.68 (2H). ^13^C NMR (75.5 MHz, CDCl_3_) δ 155.3 (C), 139.6 (C), 138.3 (C), 136.3 (C), 128.7 (CH), 128.5 (CH), 128.1 (CH), 127.7 (CH), 123.9 (CH), 88.6 (C), 73.2 (C), 67.0 (CH_2_), 47.0 (CH), 21.4 (CH_3_), 8.2 (CH_2_), −0.5 (CH); HRMS (ESI) *m*/*z*: [M + H]^+^, calcd for C_21_H_22_NO_2_ 320.1645, found 320.1642.

Benzyl (*R*)-(3-cyclopropyl-1-(2-tolyl)prop-2-yn-1-yl)carbamate (**3g**)

Compound **3g** was obtained as a white solid with a yield of 53%; m.p. 120–122 °C; [α]^D^_20_ –24.4 (c 0.98, CHCl_3_, 96% ee); enantiomeric excess (96%) was determined by chiral HPLC (Chiralcel AD-H), hexane/*^i^*PrOH 90:10, 1 mL/min, major enantiomer Rt = 11.2 min, minor enantiomer Rt = 13.2 min; ^1^H NMR (300 MHz, CDCl_3_) δ 7.54–7.51 (m, 1H), 7.37–7.30 (m, 5H), 7.20 (dd, *J* = 3.5, 5.6 Hz, 2H), 7.18–7.13 (m, 1H), 5.73 (d, *J* = 8.2 Hz, 1H), 5.16–5.07 (m, 3H), 2.38 (s, 3H), 1.31–1.22 (m, 1H), 0.80–0.73 (m, 2H), 0.72–0.65 (m, 2H). ^13^C NMR (75.5 MHz, CDCl_3_) δ 155.0 (C), 137.4 (C), 136.3 (C), 135.8 (C), 129.5 (CH), 128.5 (CH), 128.12 (CH), 128.06 (CH), 126.7 (CH), 126.2 (CH), 88.3 (C), 73.3 (C), 67.0 (CH_2_), 44.8 (CH), 19.0 (CH_3_), 8.2 (CH_2_), −0.5 (CH); HRMS (ESI) *m/z*: [M + H]^+^, calcd for C_21_H_22_NO_2_ 320.1645, found 320.1643.

Benzyl (*R*)-(1-(2-chlorophenyl)-3-cyclopropylprop-2-yn-1-yl)carbamate (**3h**)

Compound **3h** was obtained as a white solid with a yield of 30%; m.p. 110–113 °C; [α]^D^_20_ −20.0 (c 0.92, CHCl_3_, 92% ee); enantiomeric excess (92%) was determined by chiral HPLC (Chiralcel AD-H), hexane/*^i^*PrOH 90:10, 1 mL/min, major enantiomer Rt = 13.1 min, minor enantiomer Rt = 16.4 min; ^1^H NMR (300 MHz, CDCl_3_) δ 7.57–7.54 (m, 1H), 7.38–7.29 (m, 6H), 7.27–7.21 (m, 2H), 5.86 (d, *J* = 7.7 Hz, 1H), 5.33 (d, *J* = 5.9 Hz, 1H), 5.14 (d, *J* = 12.1 Hz, 1H), 5.08 (d, *J* = 12.4 Hz, 1H), 1.30–1.21 (m, 2H), 0.80–0.73 (m, 2H), 0.72–0.65 (m, 2H). ^13^C NMR (75.5 MHz, CDCl_3_) δ 154.9 (C), 136.8 (C), 136.2 (C), 133.1 (C), 130.1 (CH), 129.3 (CH), 128.8 (CH), 128.5 (CH), 128.1 (CH), 127.1 (CH), 88.7 (C), 72.2 (C), 67.0 (CH_2_), 45.4 (CH), 8.2 (CH_2_), −0.5 (CH); HRMS (ESI) *m*/*z*: [M + H]^+^, calcd for C_20_H_19_NO_2_Cl 340.1099, found 340.1086.

Benzyl (*S*)-(3-cyclopropyl-1-(naphthalen-2-yl)prop-2-yn-1-yl)carbamate (**3i**)

Compound **3i** was obtained as a white solid with a yield of 46%; m.p. 106–108 °C; [α]^D^_20_ +1.6 (c 0.87, CHCl_3_, 90% ee); enantiomeric excess (90%) was determined by chiral HPLC (Chiralcel AD-H), hexane/*^i^*PrOH 90:10, 1 mL/min, major enantiomer Rt = 16.6 min, minor enantiomer Rt = 17.5 min; ^1^H NMR (300 MHz, CDCl_3_) δ 7.93 (br.s, 1H), 7.84–7.81 (m, 3H), 7.56 (d, *J* = 8.5 Hz, 1H), 7.50–7.47 (m, 2H), 7.39–7.30 (m, 5H), 5.82 (d, *J* = 8.5 Hz, 1H), 5.31 (d, *J* = 6.8 Hz, 1H), 5.18 (d, *J* = 12.1 Hz, 1H), 5.11 (d, *J* = 12.5 Hz, 1H), 1.37–1.30 (m, 1H), 0.84–0.79 (m, 2H), 0.78–0.71 (m, 2H); ^13^C NMR (75.5 MHz, CDCl_3_) δ 155.3 (C), 137.0 (C), 136.2 (C), 133.1 (C), 133.0 (C), 128.5 (CH), 128.2 (CH), 128.1 (CH), 127.6 (CH), 126.3 (CH), 126.2 (CH), 125.6 (CH), 124.9 (CH), 89.1 (C), 73.1 (C), 67.1 (CH_2_), 47.3 (CH), 8.3 (CH_2_), −0.5 (CH); HRMS (ESI) *m*/*z*: [M + H]^+^, calcd for C_24_H_22_NO_2_ 356.1645, found 356.1645.

Benzyl (*R*)-(3-cyclopropyl-1-(furan-2-yl)prop-2-yn-1-yl)carbamate (**3j**)

Compound **3j** was obtained as an orange oil with a yield of 68%; [α]^D^_20_ +5.9 (c 1.12, CHCl_3_, 92% ee); enantiomeric excess (92%) was determined by chiral HPLC (Chiralcel AD-H), hex-ane/*^i^*PrOH 90:10, 1 mL/min, major enantiomer Rt = 14.4 min, minor enantiomer Rt = 12.7 min; ^1^H NMR (300 MHz, CDCl_3_) δ 7.37–7.33 (m, 6H), 6.32–6.31 (m, 2H), 5.69 (d, *J* = 8.6 Hz, 1H), 5.24 (d, *J* = 5.4 Hz, 1H), 5.16 (d, *J* = 12.0 Hz, 1H), 5.10 (d, *J* = 12.5 Hz, 1H), 1.31- 1.22 (m, 1H), 0.81–0.75 (m, 2H), 0.73–0.67 (m, 2H). ^13^C NMR (75.5 MHz, CDCl_3_) δ 155.1 (C), 151.6 (C), 142.6 (CH), 136.1 (C), 128.5 (CH), 128.2 (CH), 110.3 (CH), 107.2 (CH), 87.8 (C), 70.9 (C), 67.1 (CH_2_), 41.3 (CH), 8.2 (CH_2_), −0.6 (CH); HRMS (ESI) *m*/*z*: [M + H]^+^, calcd for C_18_H_18_NO_3_ 296.1281, found 296.1284.

Benzyl (*R*)-(3-cyclopropyl-1-(thiophen-2-yl)prop-2-yn-1-yl)carbamate (**3k**)

Compound **3k** was obtained as a brown solid with a yield of 64%; m.p. 91–94 °C; [α]^D^_20_ –4.8 (c 1.04, CHCl_3_, 84% ee); enantiomeric excess (84%) was determined by chiral HPLC (Chiralcel AD-H), hexane/*^i^*PrOH 90:10, 1 mL/min, major enantiomer Rt = 14.9 min, minor enantiomer Rt = 12.4 min; ^1^H NMR (300 MHz, CDCl_3_) δ 7.37–7.33 (m, 5H), 7.23 (dd, *J* = 5.1, 1.3 Hz, 1H), 7.12 (dd, *J* = 2.7 Hz, 1H), 6.94 (dd, *J* = 5.1, 3.5 Hz, 1H), 5.86 (d, *J* = 8.5 Hz, 1H), 5.29 (d, *J* = 8.1 Hz, 1H), 5.17 (d, *J* = 12.0 Hz, 1H), 5.11 (d, *J* = 12.7 Hz, 1H), 1.34- 1.24 (m, 1H), 0.83–0.77 (m, 2H), 0.76–0.70 (m, 2H). ^13^C NMR (75.5 MHz, CDCl_3_) δ 155.1 (C), 143.7 (C), 136.1 (C), 128.5 (CH), 128.2 (CH), 128.1 (CH), 126.7(CH), 125.5 (CH), 125.3 (CH), 88.3 (C), 72.7 (C), 67.1 (CH_2_), 42.9 (CH), 8.19 (CH_2_), 8.17 (CH_2_), −0.6 (CH); HRMS (ESI) *m*/*z*: [M + H]^+^, calcd for C_18_H_18_NO_2_S 312.1053, found 312.1054.

Benzyl (*R*)-(3-cyclopropyl-1-(furan-3-yl)prop-2-yn-1-yl)carbamate (**3l**)

Compound **3l** was obtained as an orange oil with a yield of 52%; [α]^D^_20_ –11.0 (c 1.00, CHCl_3_, 69% ee); enantiomeric excess (69%) was determined by chiral HPLC (Chiralcel AD-H), hexane/*^i^*PrOH 90:10, 1 mL/min, major enantiomer Rt = 13.6 min, minor enantiomer Rt = 11.8 min; ^1^H NMR (300 MHz, CDCl_3_) δ 7.37 (s, 1H), 7.29–7.26 (m, 6H), 6.33 (s, 1H), 5.47 (d, *J* = 7.7 Hz, 1H), 5.10–5.01 (m, 3H), 1.24–1.14 (m, 1H), 0.74–0.68 (m, 2H), 0.63–0.58 (m, 2H). ^13^C NMR (75.5 MHz, CDCl_3_) δ 155.3 (C), 143.5 (CH), 140.2 (CH), 136.2 (C), 128.5 (CH), 128.2 (CH), 128.1 (CH), 125.3 (C), 109.3 (CH), 87.2 (C), 72.6 (C), 67.1 (CH_2_), 39.5 (CH), 8.2 (CH_2_), −0.7 (CH); HRMS (ESI) *m*/*z*: [M + H]^+^, calcd for C_18_H_18_NO_3_ 296.1281, found 296.1285,

Benzyl (*S*)-(1-cyclopropyl-5-phenylpent-1-yn-3-yl)carbamate (**3m**)

Compound **3m** was obtained as a yellow oil with a yield of 59%; [α]^D^_20_ −6.6 (c 0.99, CHCl_3_, 42% ee); enantiomeric excess (42%) was determined by chiral HPLC (Chiralcel AD-H), hexane/*^i^*PrOH 90:10, 1 mL/min, major enantiomer Rt = 11.7 min, minor enantiomer Rt = 13.1 min; ^1^H NMR (300 MHz, CDCl_3_) δ 7.37–7.26 (m, 7H), 7.21–7.16 (m, 3H), 5.10 (s, 2H), 4.89 (d, *J* = 7.7 Hz, 1H), 4.46 (q, *J* = 7.7 Hz, 1H), 2.76–2.70 (m, 2H), 2.04–1.87 (m, 2H), 1.29–1.19 (m, 1H), 0.80–0.74 (m, 2H), 0.69–0.63 (2H). ^13^C NMR (75.5 MHz, CDCl_3_) δ 155.3 (C), 141.1 (C), 136.3 (C), 128.5 (CH), 128.4 (CH), 128.1 (CH), 126.0 (CH), 87.4 (C), 74.0 (C), 66.8 (CH_2_), 43.5 (CH), 38.1 (CH_2_), 31.9 (CH_2_), 8.20 (CH_2_), 8.19 (CH_2_), −0.7 (CH); HRMS (ESI) *m*/*z*: [M + H]^+^, calcd for C_22_H_24_NO_2_ 334.1802, found 334.1797.

Benzyl (*S*)-(1-cyclopropylhept-1-yn-3-yl)carbamate (**3n**)

Compound **3n** was obtained as a colorless oil with a yield of 50%; [α]^D^_20_ –15.6 (c 1.00, CHCl_3_, 43% ee); enantiomeric excess (43%) was determined by chiral HPLC (Chiralcel IC), hex-ane/*^i^*PrOH 95:5, 1 mL/min, major enantiomer Rt = 9,6 min, minor enantiomer Rt = 9.2 min; ^1^H NMR (300 MHz, CDCl_3_) δ 7.36–7.31 (m, 5H), 5.10 (s, 2H), 5.84 (d, *J* = 5.7 Hz, 1H), 4.40 (q, *J* = 10.5 Hz, 1H), 1.66–1.55 (m, 2H), 1.40–1.25 (m, 4H), 1.22–1.16 (m, 1H), 0.90 (t, *J* = 13.9 Hz, 3H), 0.77–0.71 (m, 2H), 0.66–0.60 (m, 2H). ^13^C NMR (75.5 MHz, CDCl_3_) δ 155.3 (C), 136.4 (C), 128.5 (CH), 128.1 (CH), 86.7 (C), 74.6 (C), 66.8 (CH_2_), 43.7 (CH), 36.3 (CH_2_), 27.7 (CH_2_), 22.2 (CH_2_), 14.0 (CH_3_), 8.14 (CH_2_), 8.13 (CH_2_), -0.7 (CH); HRMS (ESI) *m*/*z*: [M + H]^+^, calcd for C_18_H_24_NO_2_ 286.1802, found 286.1802.

Benzyl (*S*)-(1-cyclohexyl-3-cyclopropylprop-2-yn-1-yl)carbamate (**3o**)

Compound **3o** was obtained as a pale yellow solid with a yield of 63%; m.p. 101–104 °C; [α]^D^_20_ −23.6 (c 0.90, CHCl_3_, 65% ee); enantiomeric excess (65%) was determined by chiral HPLC (Chiralcel IC), hexane/*^i^*PrOH 98:2, 1 mL/min, major enantiomer Rt = 15.7 min, minor enantiomer Rt = 16.9 min; ^1^H NMR (300 MHz, CDCl_3_) δ 7.37–7.34 (m, 5H), 5.12 (d, *J* = 12.1 Hz, 1H), 5.07 (d, *J* = 12.1 Hz, 1H), 4.86 (d, *J* = 7.4 Hz, 1H), 4.29 (d, *J* = 7.0 Hz, 1H), 1.76–1.63 (m, 5H), 1.25–1.03 (m, 7H), 0.77–0.69 (m, 2H), 0.66–0.60 (m, 2H). ^13^C NMR (75.5 MHz, CDCl_3_) δ 155.5 (C), 136.4 (C), 128.5 (CH), 128.1 (CH), 87.5 (C), 73.2 (C), 66.8 (CH2), 48.8 (CH), 42.9 (CH), 29.2 (CH_2_), 28.3 (CH_2_), 26.2 (CH_2_), 25.9 (CH), 25.8 (CH), 8.2 (CH_2_), −0.6 (CH); HRMS (ESI) *m*/*z*: [M + H]^+^, calcd for C_20_H_26_NO_2_ 312.1958, found 312.1959.

## 4. Conclusions

In summary, we have developed a simple and convenient method for the synthesis of *N*-carbamoyl-protected propargylic amines substituted with a cyclopropyl group. Although related reactions with α-amido sulfones and phenylacetylene and other arylacetylenes have been reported, this is the first example using cyclopropylacetylene. The reaction is catalyzed by a chiral BINOL-type zinc complex and provides the corresponding products in good yields and enantioselectivities for a number of α-amido sulfones with the best results being obtained with the *ortho*-methylphenyl derivative.

## Data Availability

Not applicable.

## Supplementary Materials

The following supporting information can be downloaded at: https://www.mdpi.com/article/10.3390/molecules27123763/s1, ^1^H-NMR, ^13^C-NMR and HPLC traces of compounds **3a**–**o** are available online.

## Sample Availability

Samples of the compounds are not available from the authors.
