# Supplementary material for: Catalytic Enantioselective Cyclopropylalkynylation of Aldimines Generated In Situ from α-Amido Sulfones
Source: Molecules. 2022 Jun 11;27(12):3763. doi: 10.3390/molecules27123763 (PMC9231313; doi:10.3390/molecules27123763)
Supplement: Supplementary file 1 [file molecules-27-03763-s001.zip › molecules-1763345-supplementary.pdf]

Catalytic enantioselective cyclopropylalkynylation of aldimines generated in situ from α-amido sulfones

Alicia Monleón [1], Gonzalo Blay [1,*] and José R. Pedro [1,*]

[1] Departament de Química Orgànica, Facultad de Química, Universitat de València, C/ Dr Moliner 50, 46100-Burjassot, Valencia, Spain; alicia.monleon@uv.es
* Correspondence: gonzalo.blay@uv.es; jose.r.pedro@uv.es

SUPPORTING INFORMATION

Table of Contents:

^{1}H NMR and ^{13}C NMR spectra S2-S31
Chiral analysis chromatograms S32-S46

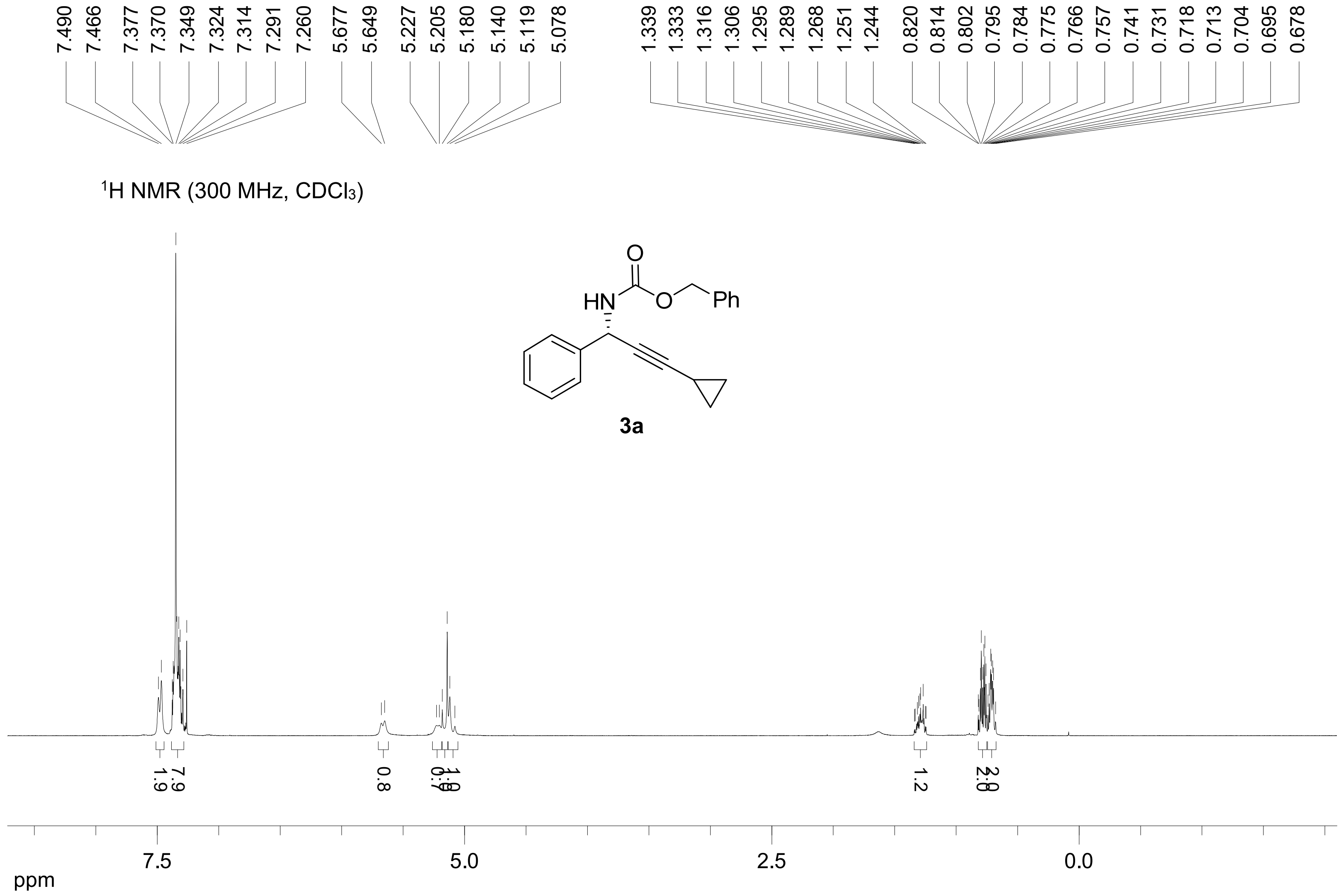
7.490 7.466 7.377 7.370 7.349 7.324 7.314 7.291 7.260 5.677 5.649 5.227 5.205 5.180 5.140 5.119 5.078
1.339 1.333 1.316 1.306 1.295 1.289 1.268 1.251 1.244 0.820 0.814 0.802 0.795 0.784 0.775 0.766 0.757 0.741 0.731 0.718 0.713 0.704 0.695 0.678
^{1}H NMR (300 MHz, $CDCl_3$)
HN
O
O
Ph
3a
1.9 7.9 0.8 0.7 1.0 1.2 2.0 2.0
7.5
5.0
2.5
0.0
ppm

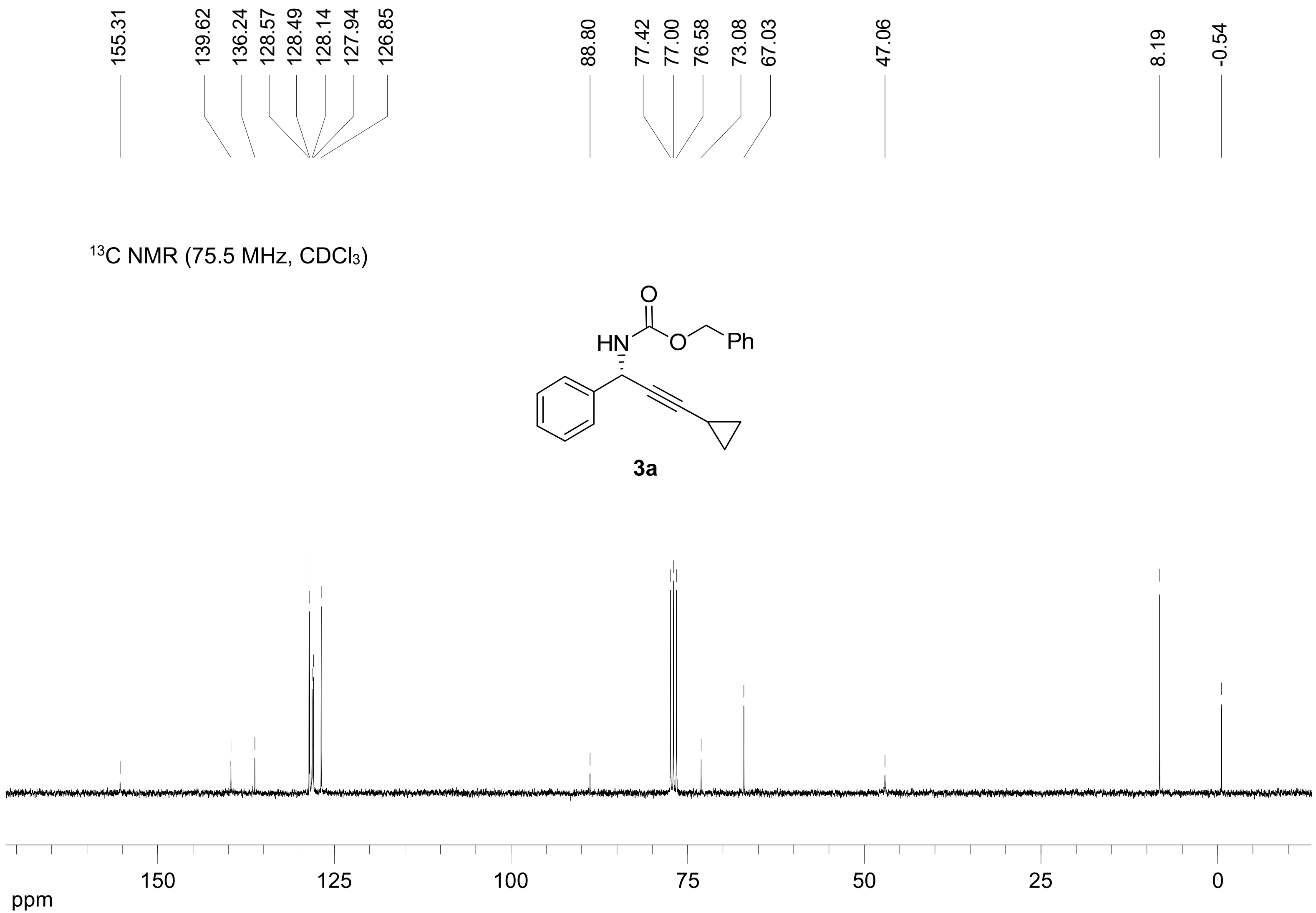
155.31
139.62
136.24
128.57
128.49
128.14
127.94
126.85
88.80
77.42
77.00
76.58
73.08
67.03
47.06
8.19
-0.54
^{13}C NMR (75.5 MHz, $CDCl_3$)
O
HN
O
Ph
3a
150
125
100
75
50
25
0
ppm

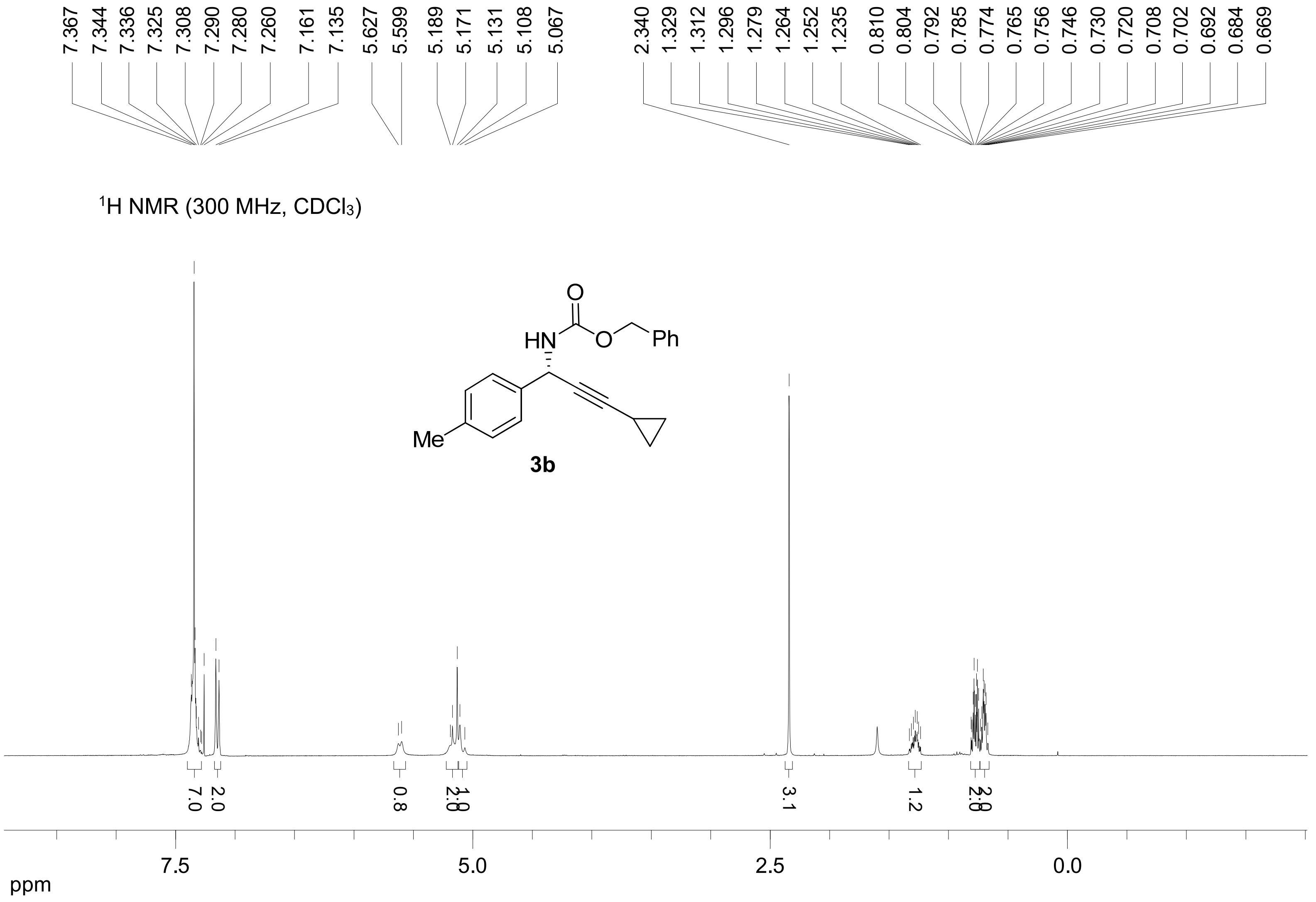
7.367 7.344 7.336 7.325 7.308 7.290 7.280 7.260 7.161 7.135 5.627 5.599 5.189 5.171 5.131 5.108 5.067
2.340 1.329 1.312 1.296 1.279 1.264 1.252 1.235 0.810 0.804 0.792 0.785 0.774 0.765 0.756 0.746 0.730 0.720 0.708 0.702 0.692 0.684 0.669
^{1}H NMR (300 MHz, CDCl$_3$)
O
HN
O
Ph
Me
3b
7.0 2.0 0.8 2.0 1.0 3.1 1.2 2.0 2.0
7.5
5.0
2.5
0.0
ppm

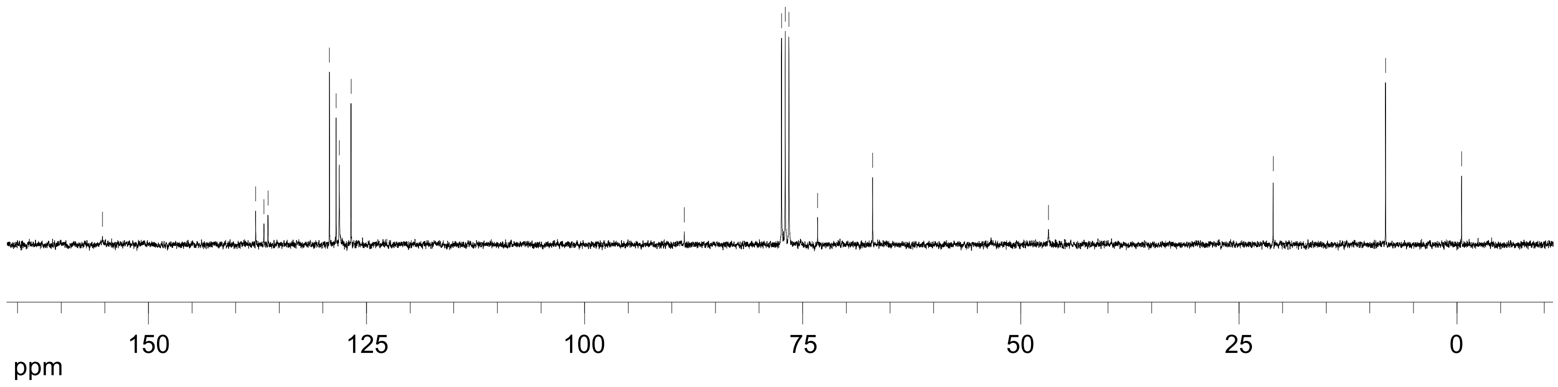

^{13}C NMR (75.5 MHz, $CDCl_3$)

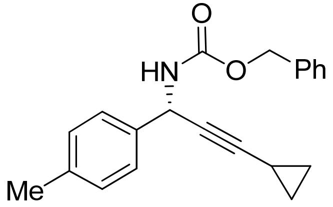

3b

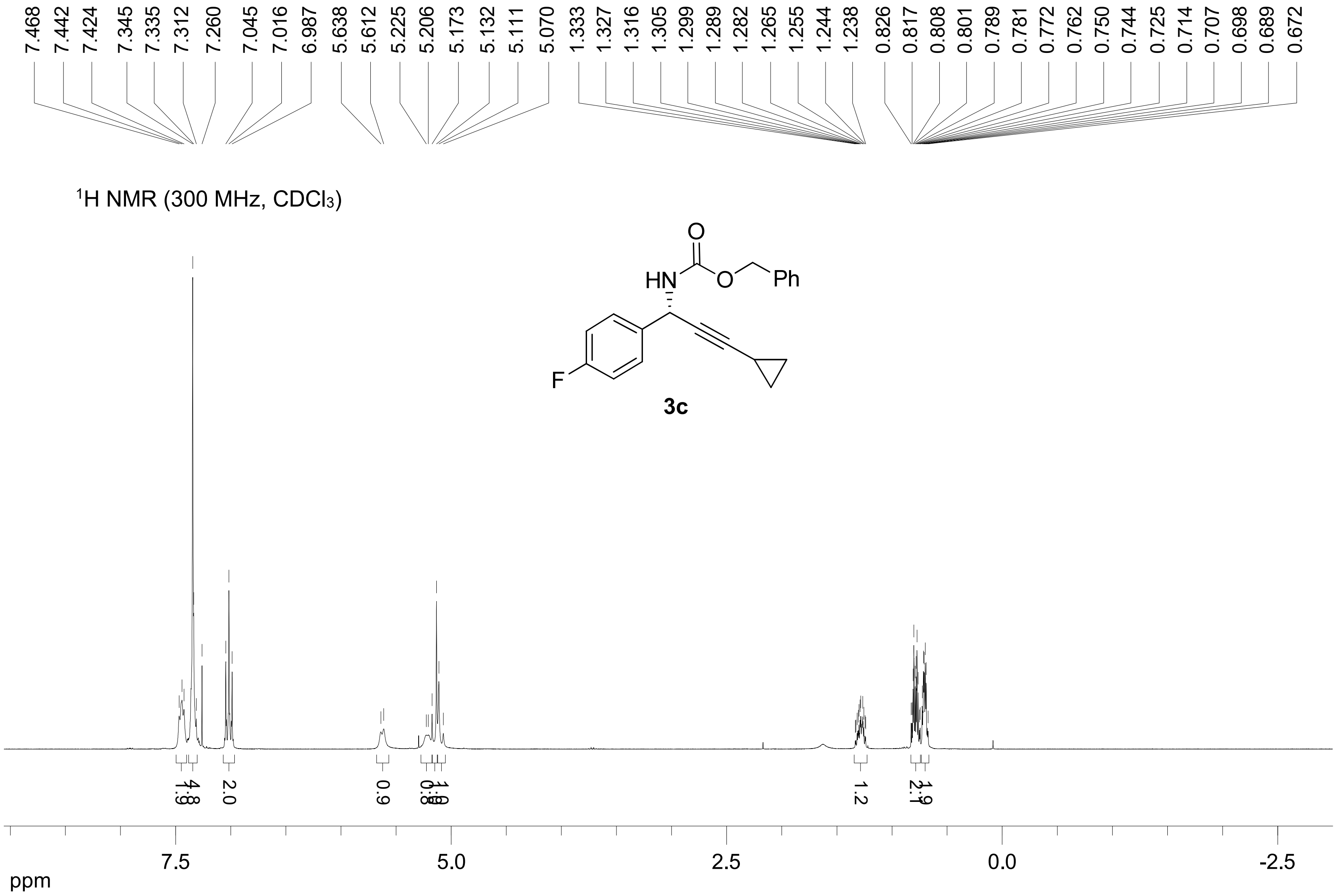

7.468 7.442 7.424 7.345 7.335 7.312 7.260 7.045 7.016 6.987 5.638 5.612 5.225 5.206 5.173 5.132 5.111 5.070 1.333 1.327 1.316 1.305 1.299 1.289 1.282 1.265 1.255 1.244 1.238 0.826 0.817 0.808 0.801 0.789 0.781 0.772 0.762 0.750 0.744 0.725 0.714 0.707 0.698 0.689 0.672
^{1}H NMR (300 MHz, CDCl$_3$)
O
HN
O
Ph
F
3c
1.9
4.8
2.0
0.9
0.8
1.0
1.2
2.1
1.9
7.5
5.0
2.5
0.0
-2.5
ppm

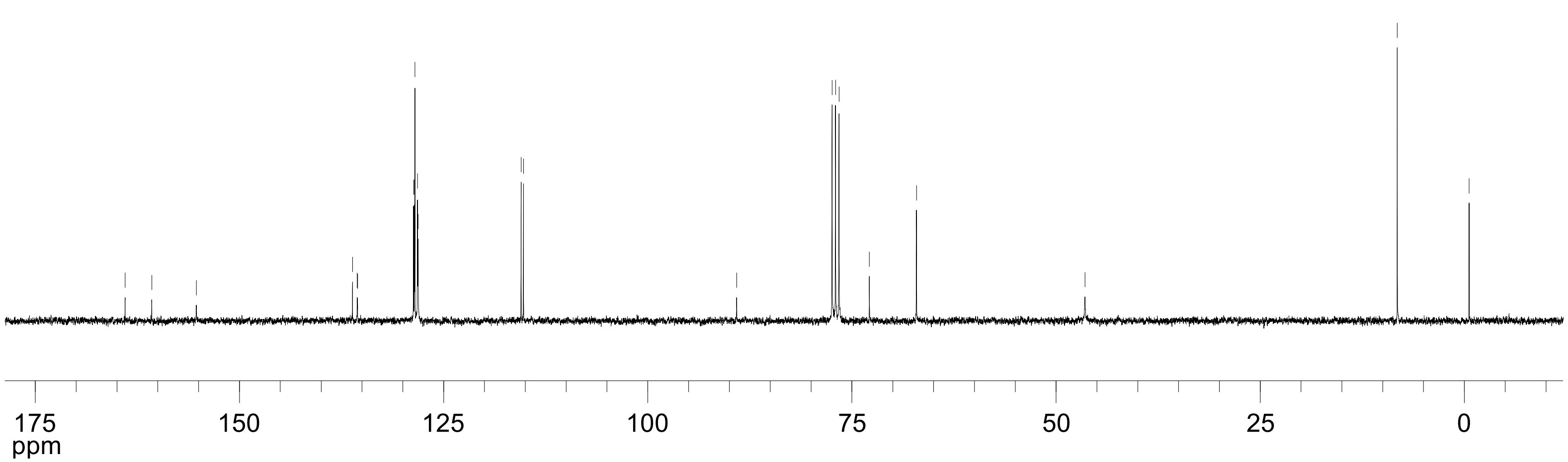

^{13}C NMR (75.5 MHz, $CDCl_3$)

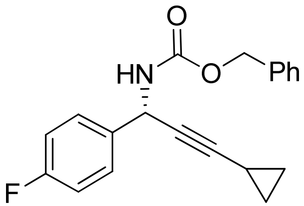

3c

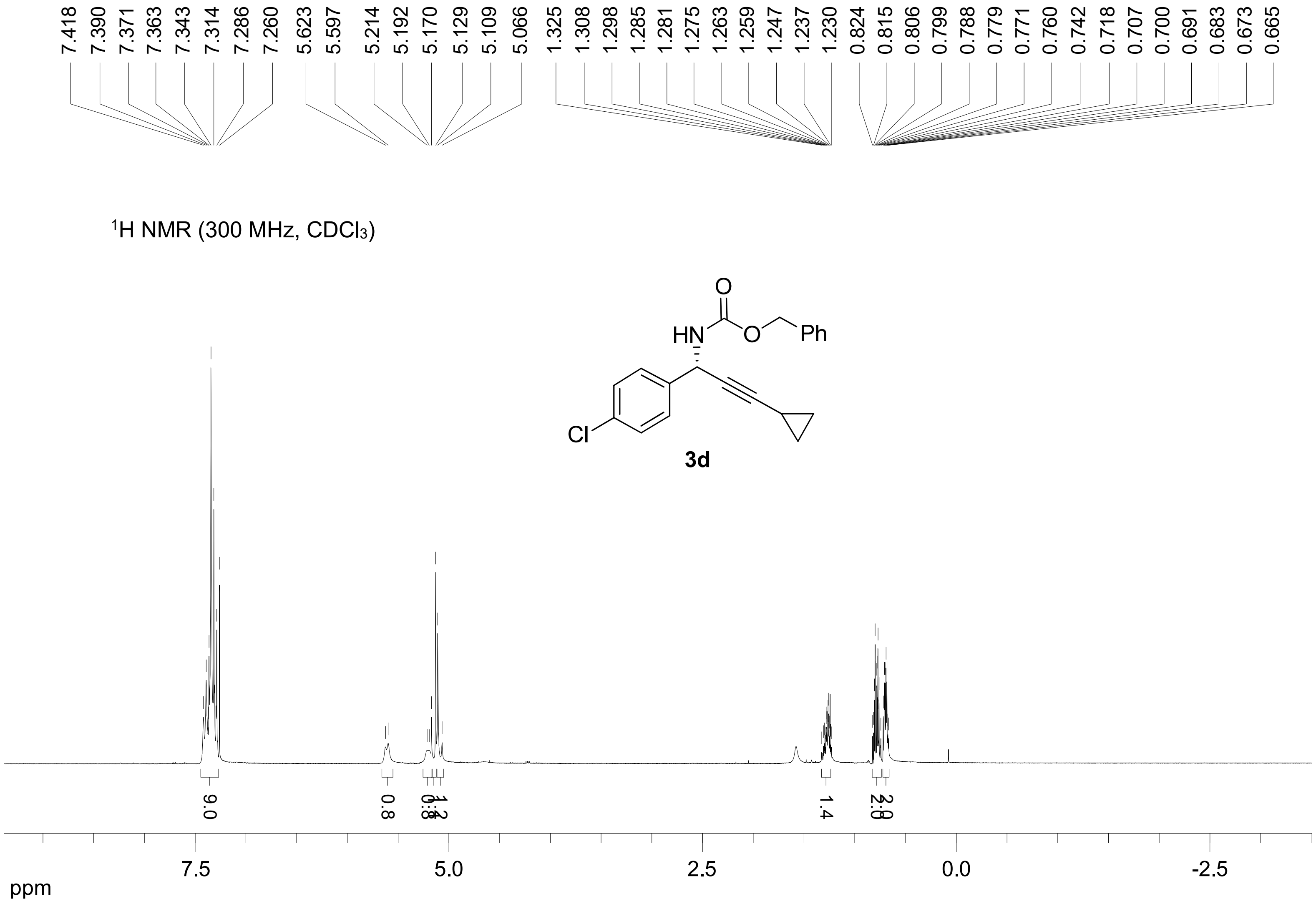
7.418 7.390 7.371 7.363 7.343 7.314 7.286 7.260 5.623 5.597 5.214 5.192 5.170 5.129 5.109 5.066 1.325 1.308 1.298 1.285 1.281 1.275 1.263 1.259 1.247 1.237 1.230 0.824 0.815 0.806 0.799 0.788 0.779 0.771 0.760 0.742 0.718 0.707 0.700 0.691 0.683 0.673 0.665
1H NMR (300 MHz, CDCl3)
O
HN
O
Ph
Cl
3d
9.0
0.8
0.8
1.2
1.4
2.0
2.0
7.5
5.0
2.5
0.0
-2.5
ppm

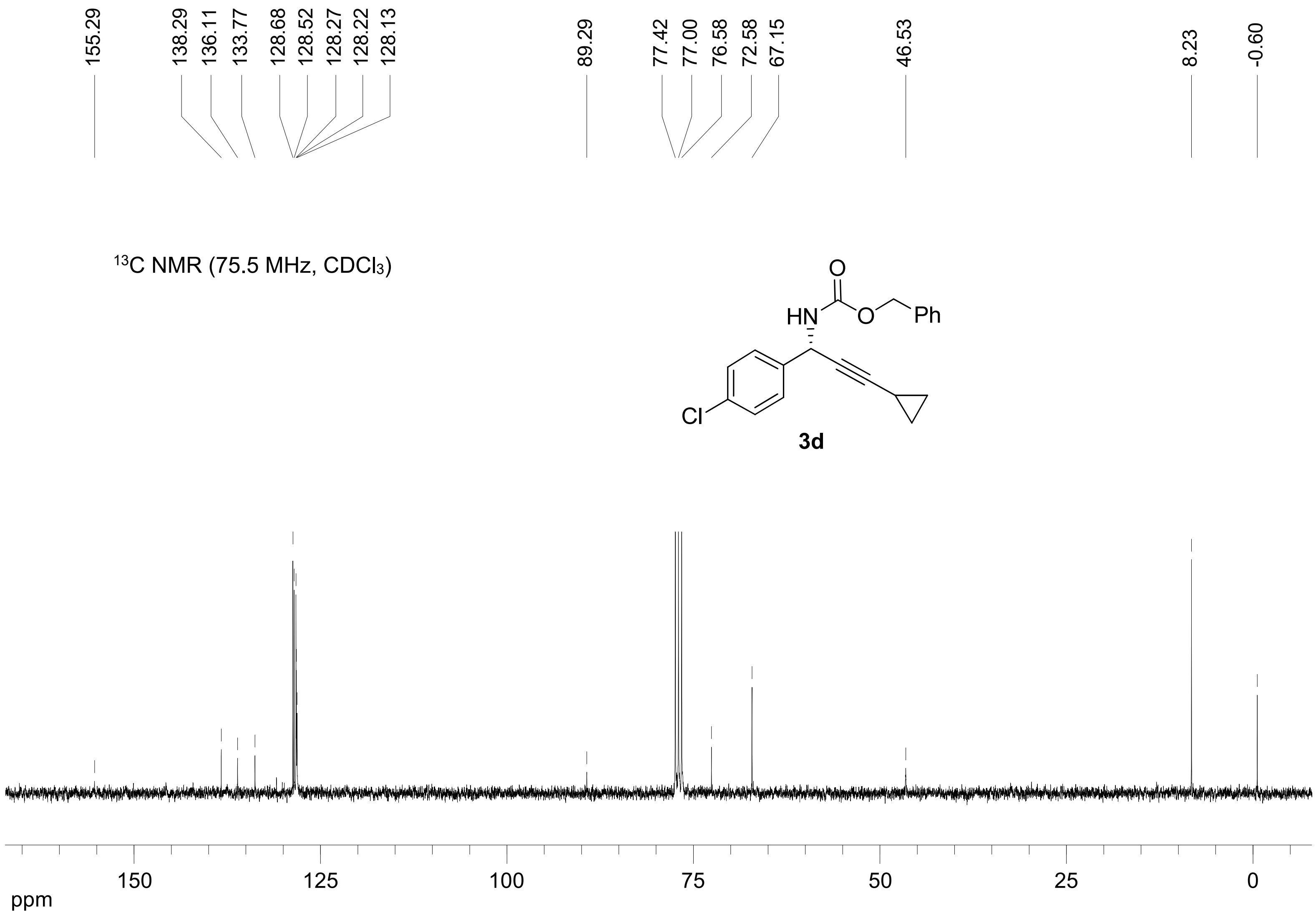

^{13}C NMR (75.5 MHz, $CDCl_3$)

3d

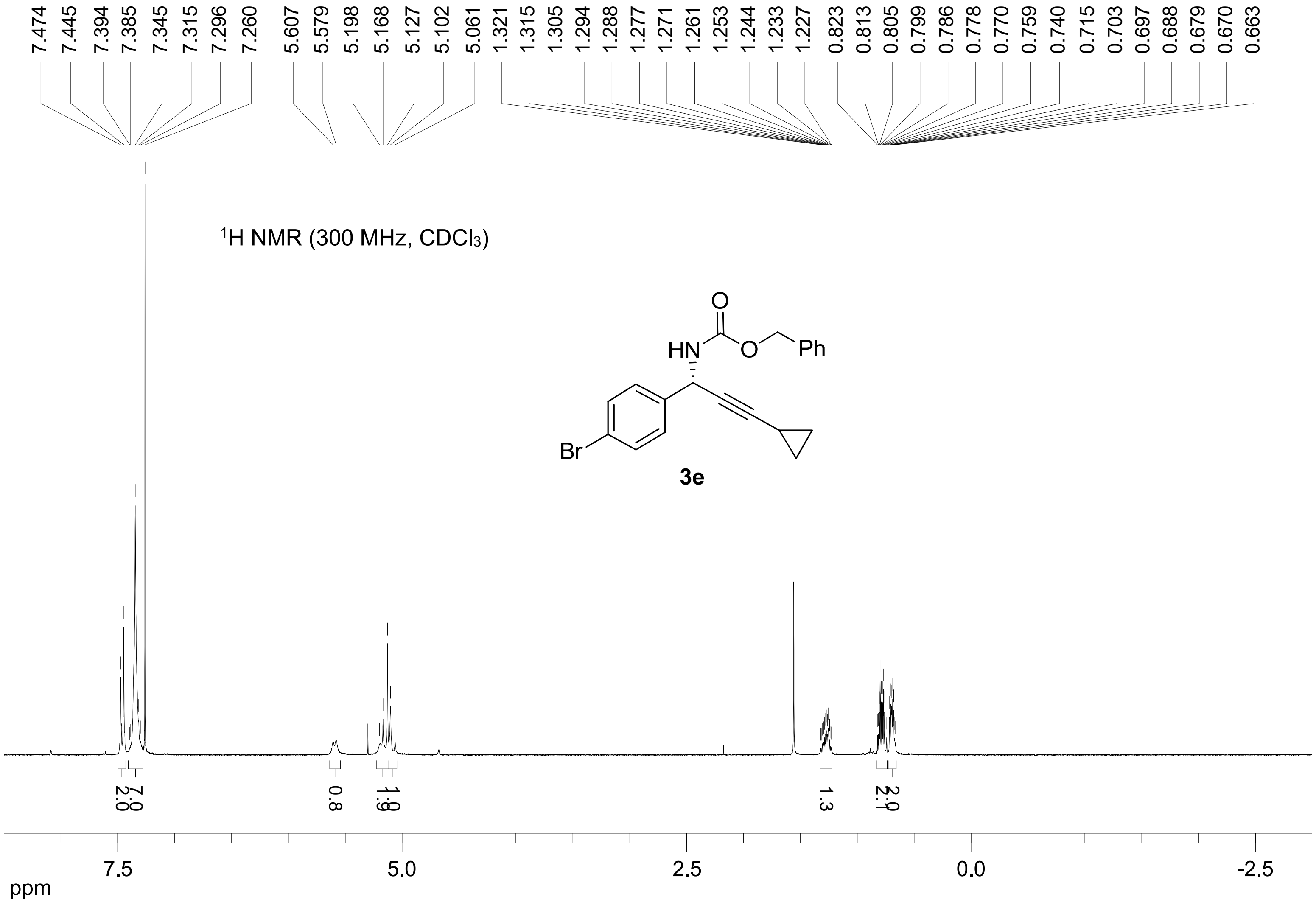
7.474 7.445 7.394 7.385 7.345 7.315 7.296 7.260 5.607 5.579 5.198 5.168 5.127 5.102 5.061 1.321 1.315 1.305 1.294 1.288 1.277 1.271 1.261 1.253 1.244 1.233 1.227 0.823 0.813 0.805 0.799 0.786 0.778 0.770 0.759 0.740 0.715 0.703 0.697 0.688 0.679 0.670 0.663
1H NMR (300 MHz, CDCl3)
O
HN
O
Ph
Br
3e
2.0 7.0 0.8 1.9 1.0 1.3 2.1 2.0
7.5 5.0 2.5 0.0 -2.5
ppm

^{13}C NMR (75.5 MHz, $CDCl_3$)

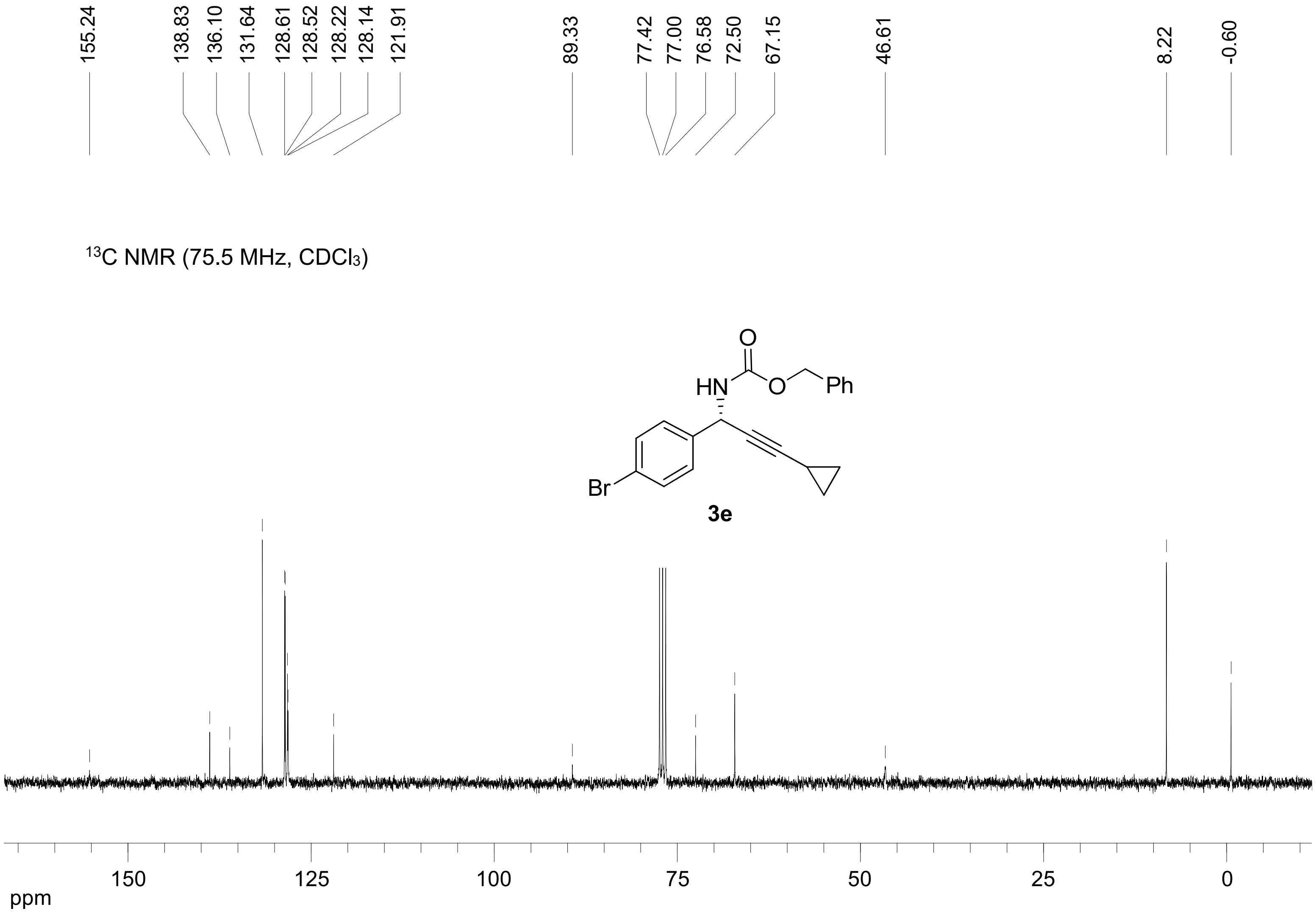

3e

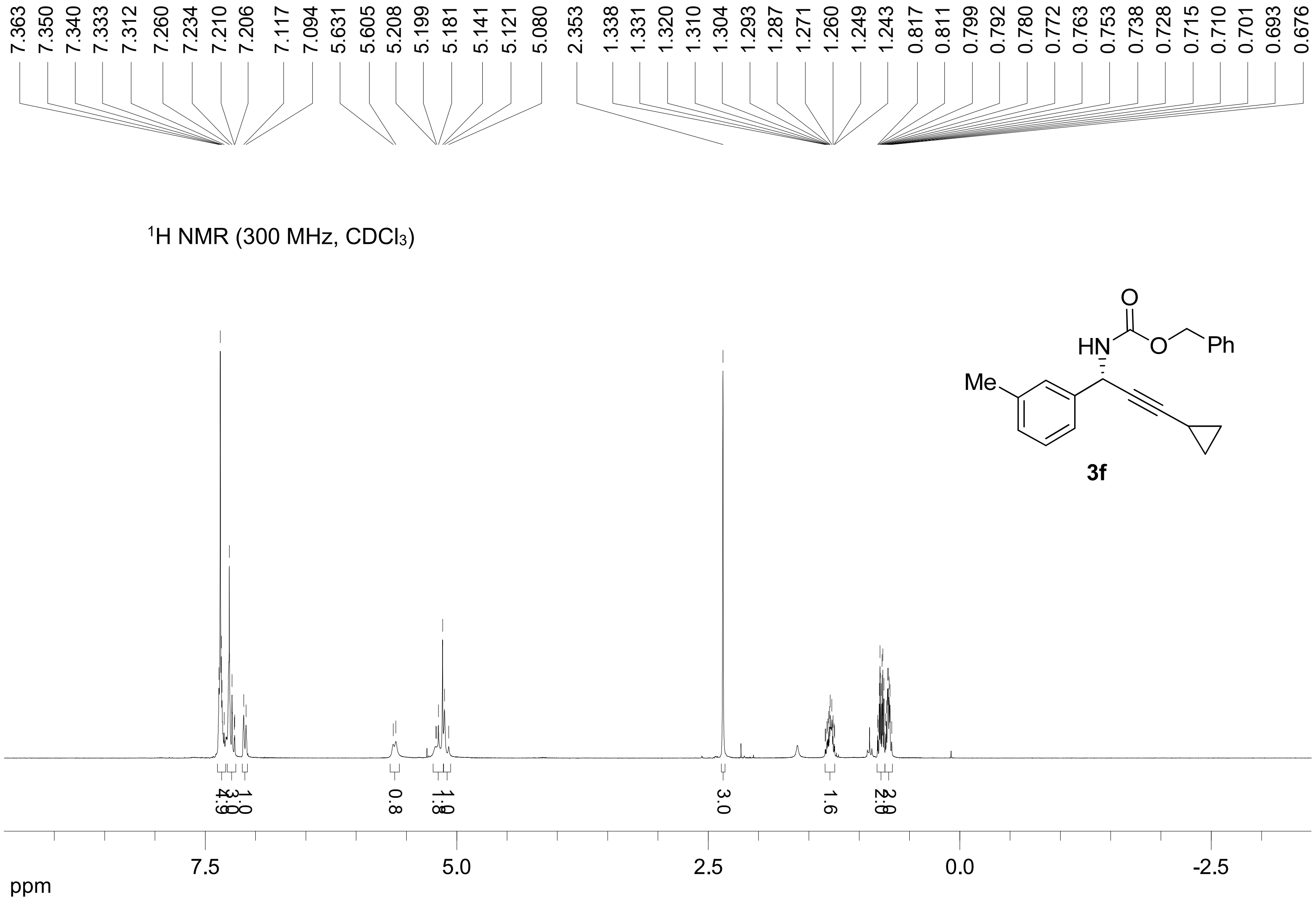
7.363
7.350
7.340
7.333
7.312
7.260
7.234
7.210
7.206
7.117
7.094
5.631
5.605
5.208
5.199
5.181
5.141
5.121
5.080
2.353
1.338
1.331
1.320
1.310
1.304
1.293
1.287
1.271
1.260
1.249
1.243
0.817
0.811
0.799
0.792
0.780
0.772
0.763
0.753
0.738
0.728
0.715
0.710
0.701
0.693
0.676
¹H NMR (300 MHz, CDCl₃)
O
HN
O
Ph
Me
3f
4.9
3.0
1.0
0.8
1.8
1.0
3.0
1.6
2.0
2.0
7.5
5.0
2.5
0.0
-2.5
ppm

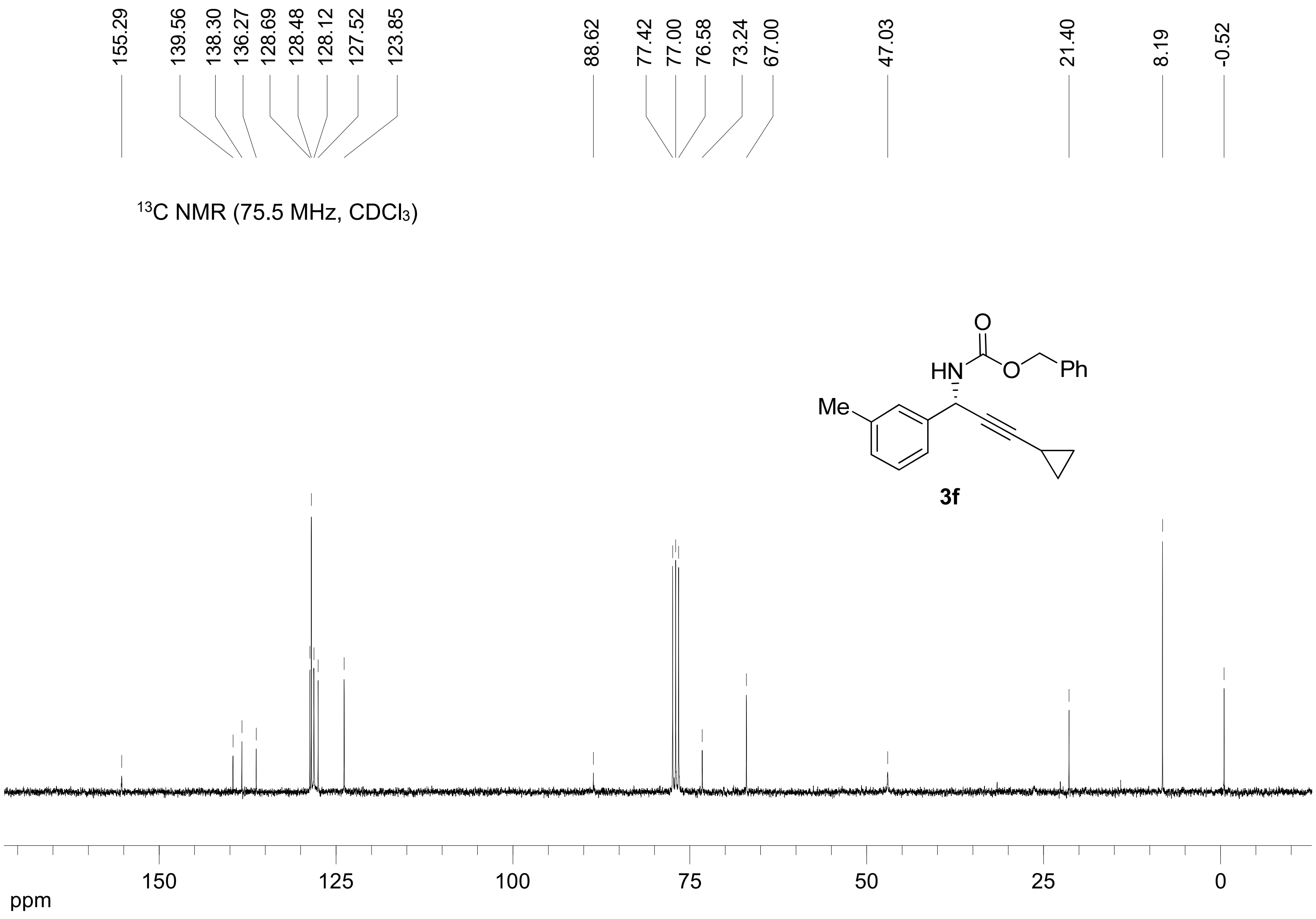
155.29
139.56
138.30
136.27
128.69
128.48
128.12
127.52
123.85
88.62
77.42
77.00
76.58
73.24
67.00
47.03
21.40
8.19
-0.52
^{13}C NMR (75.5 MHz, $CDCl_3$)
O
HN
O
Ph
Me
3f
150
125
100
75
50
25
0
ppm

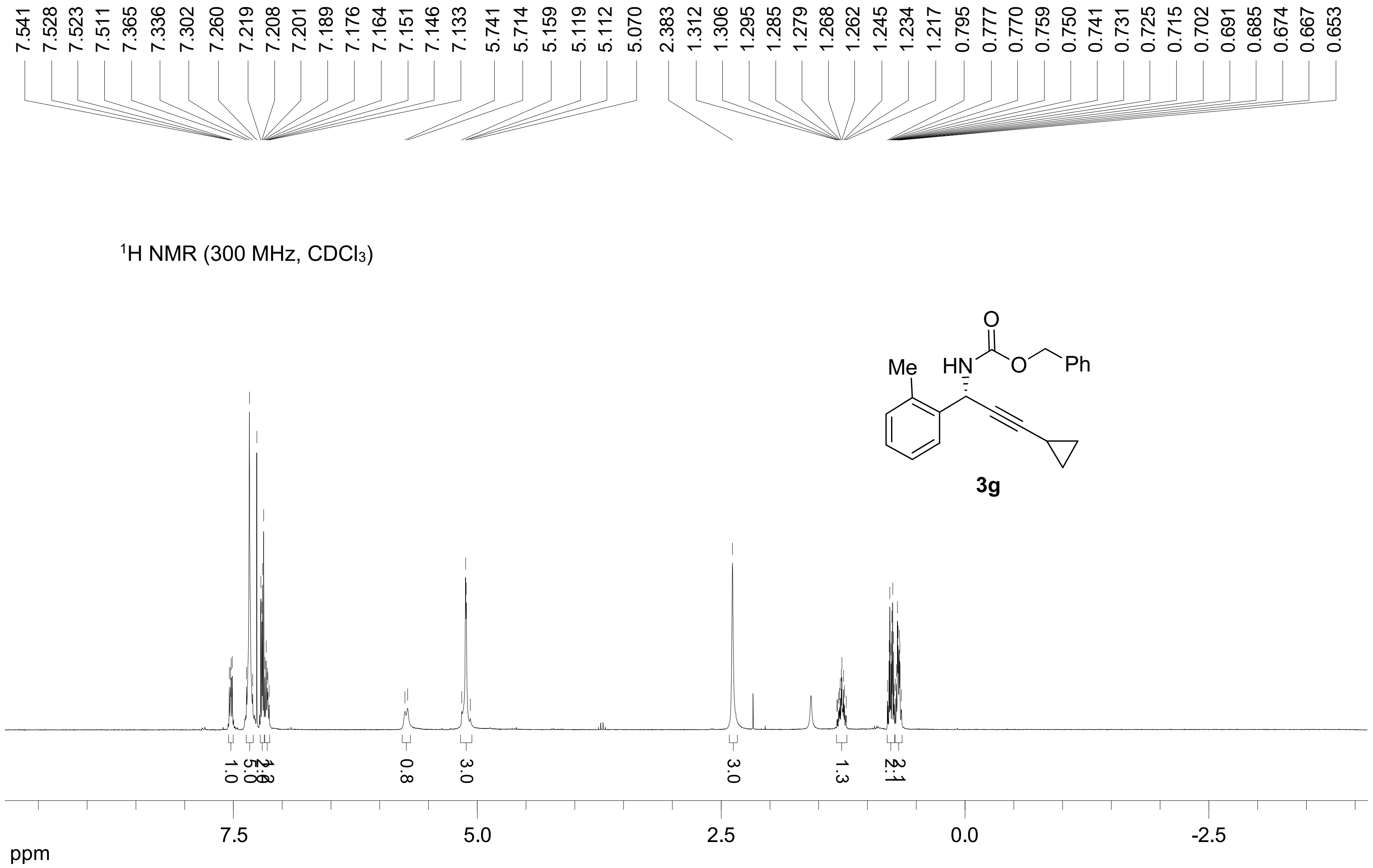
7.541 7.528 7.523 7.511 7.365 7.336 7.302 7.260 7.219 7.208 7.201 7.189 7.176 7.164 7.151 7.146 7.133 5.741 5.714 5.159 5.119 5.112 5.070 2.383 1.312 1.306 1.295 1.285 1.279 1.268 1.262 1.245 1.234 1.217 0.795 0.777 0.770 0.759 0.750 0.741 0.731 0.725 0.715 0.702 0.691 0.685 0.674 0.667 0.653
1H NMR (300 MHz, CDCl3)
Me
HN
O
O
Ph
3g
1.0
0.8
3.0
3.0
1.3
2.1
7.5
5.0
2.5
0.0
-2.5
ppm

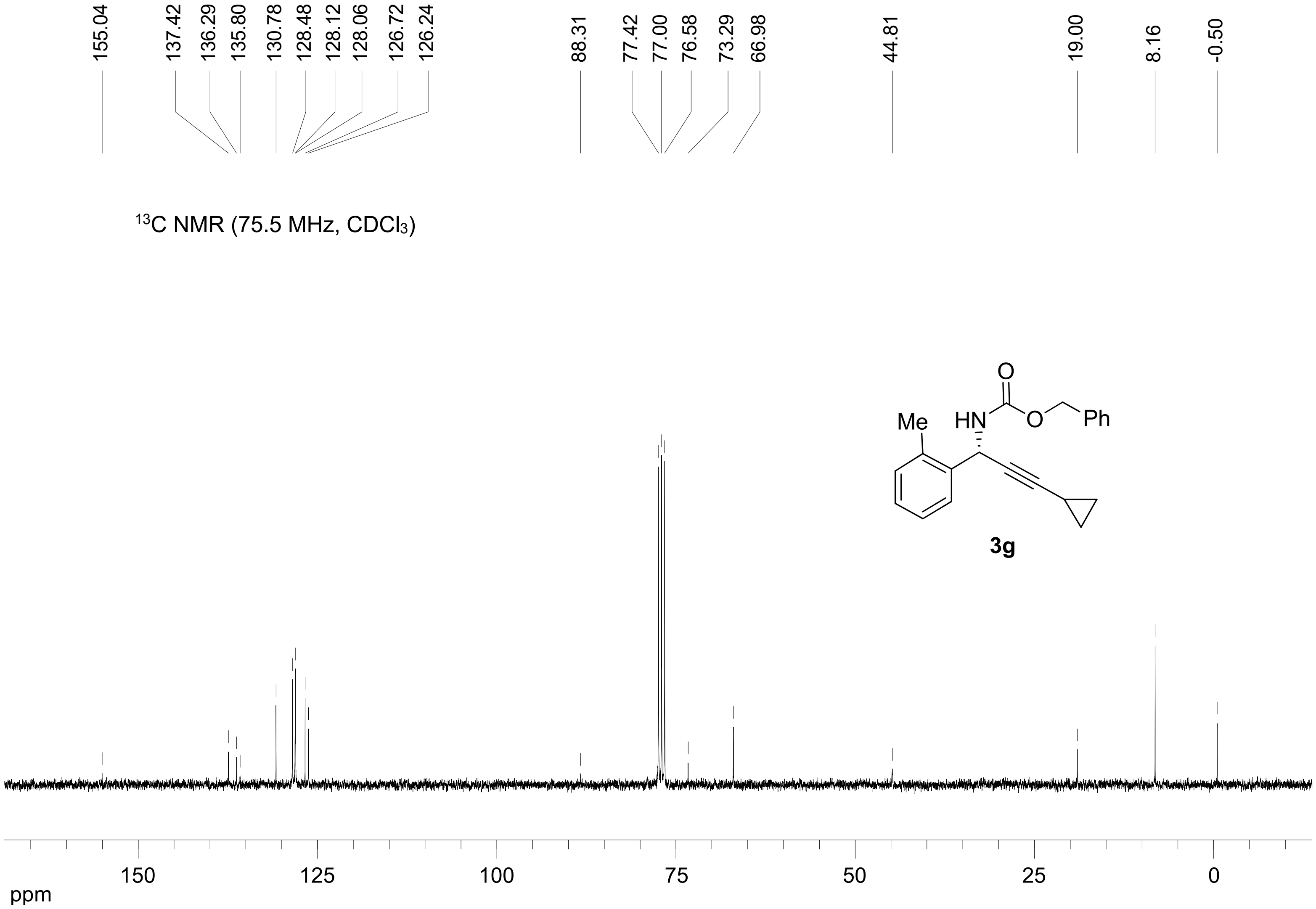

^{13}C NMR (75.5 MHz, $CDCl_3$)

3g

^{1}H NMR (300 MHz, $CDCl_3$)

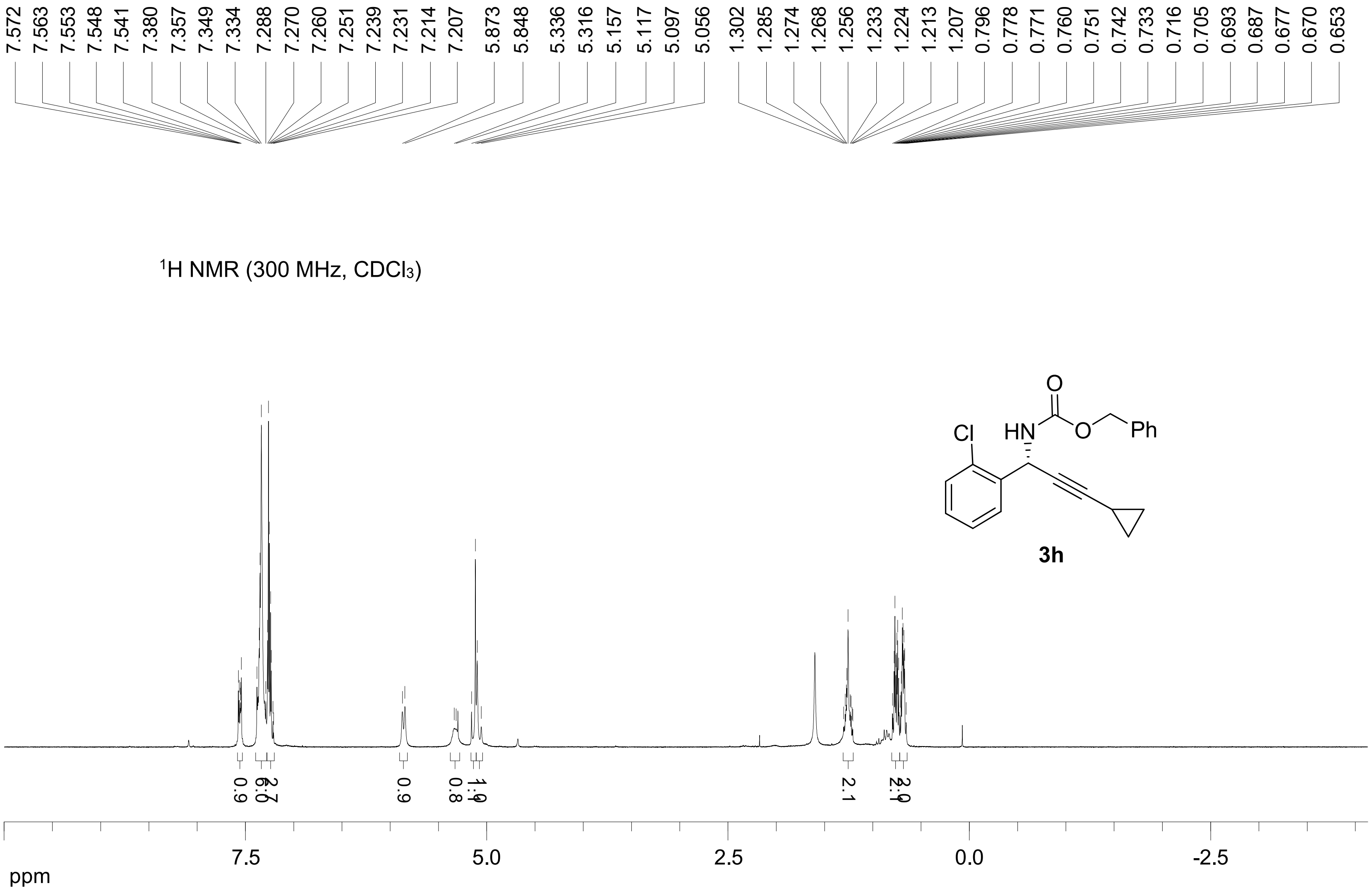

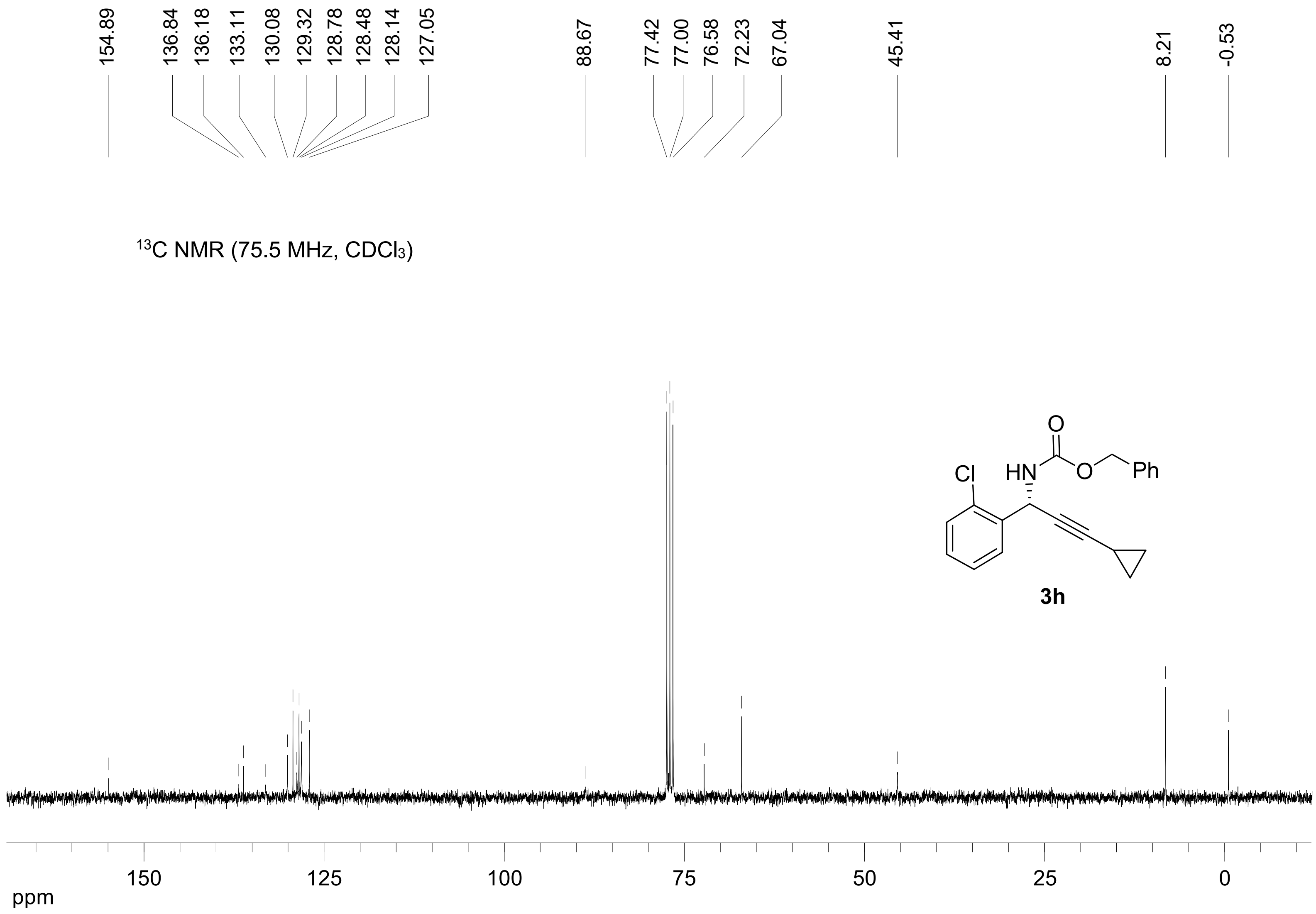
154.89
136.84
136.18
133.11
130.08
129.32
128.78
128.48
128.14
127.05
88.67
77.42
77.00
76.58
72.23
67.04
45.41
8.21
-0.53
13C NMR (75.5 MHz, CDCl3)
O
HN
O
Ph
Cl
3h
150
125
100
75
50
25
0
ppm

^{1}H NMR (300 MHz, $CDCl_3$)

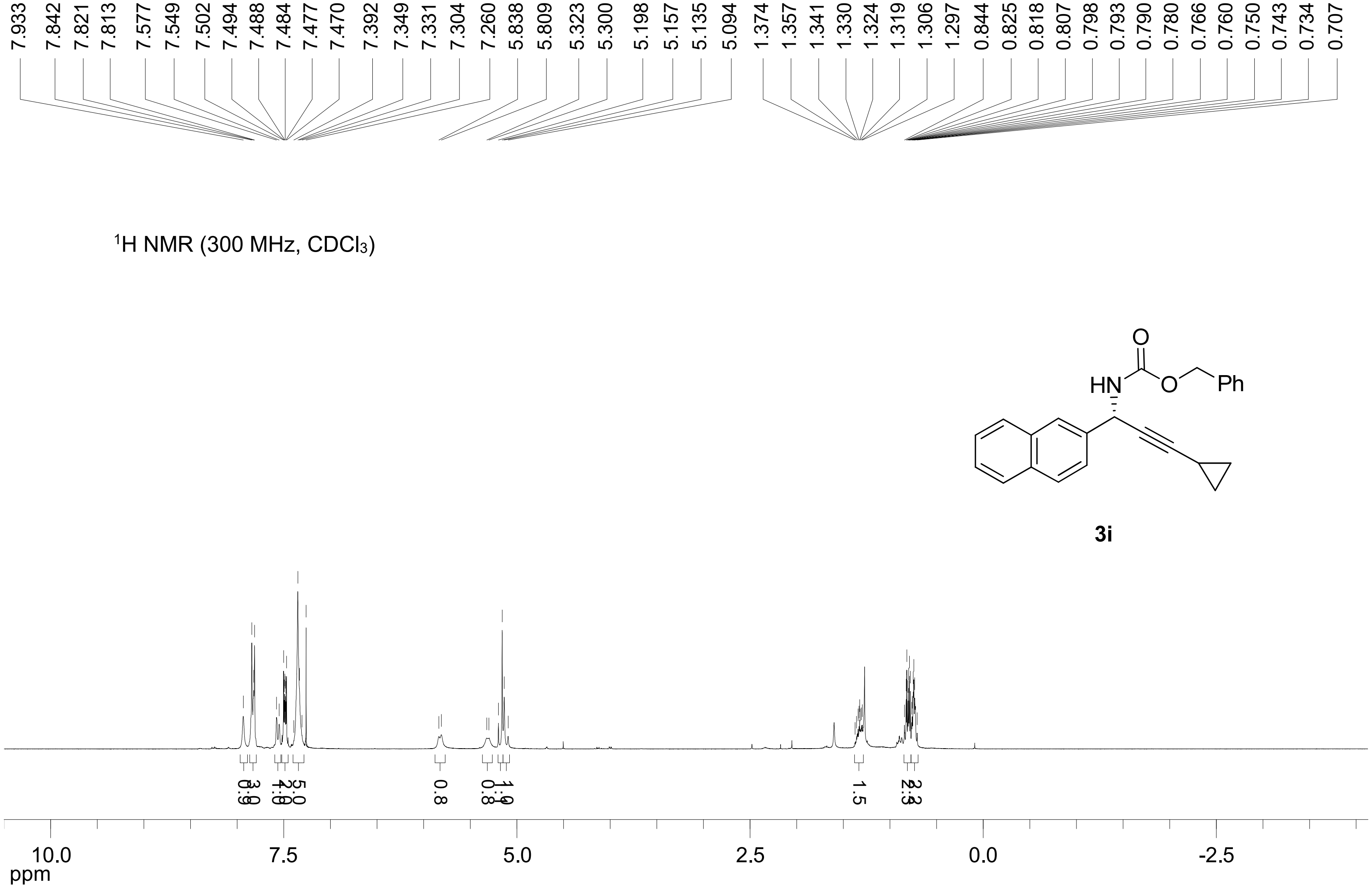

3i

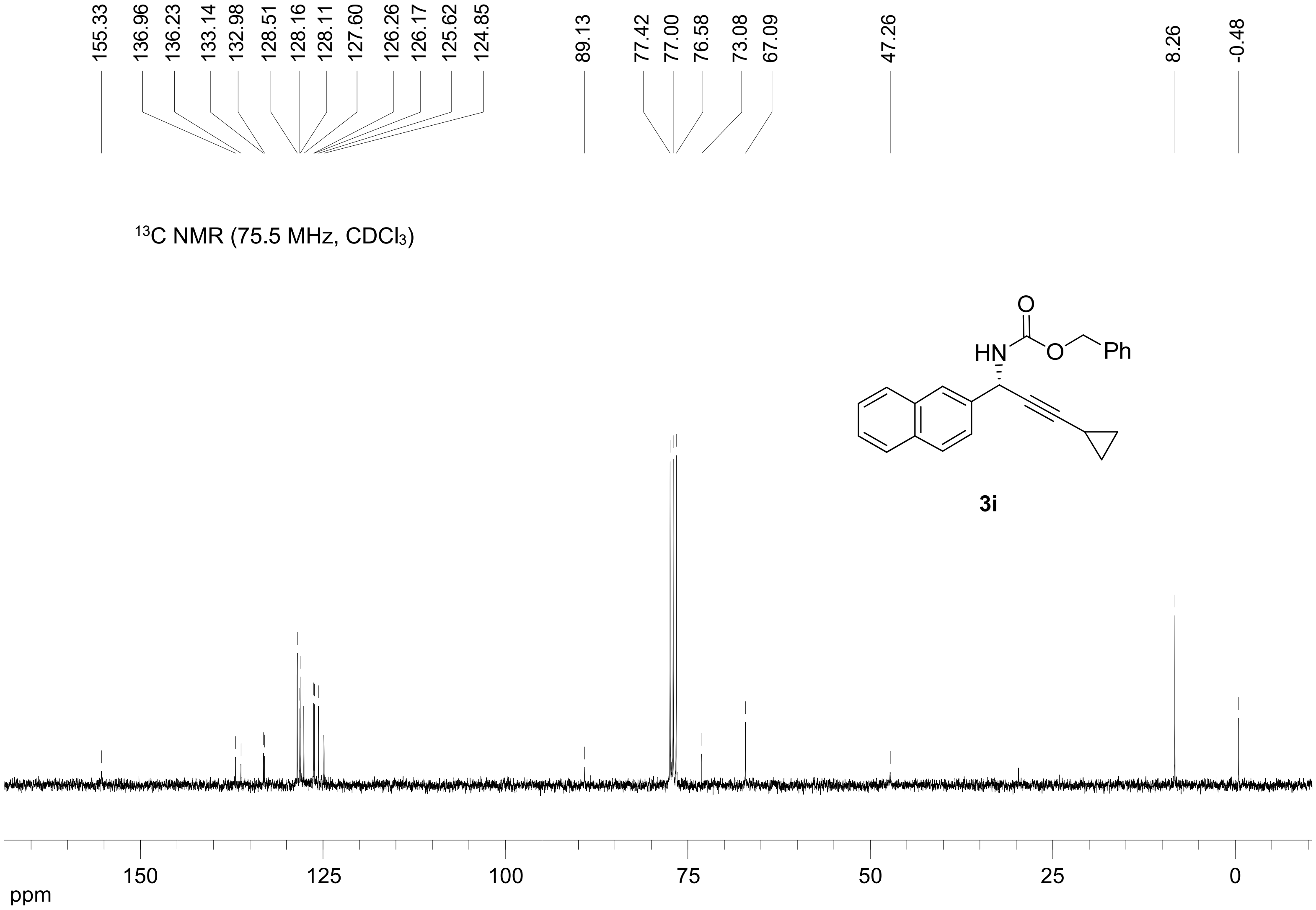

^{13}C NMR (75.5 MHz, $CDCl_3$)

3i

^1H NMR (300 MHz, $CDCl_3$)

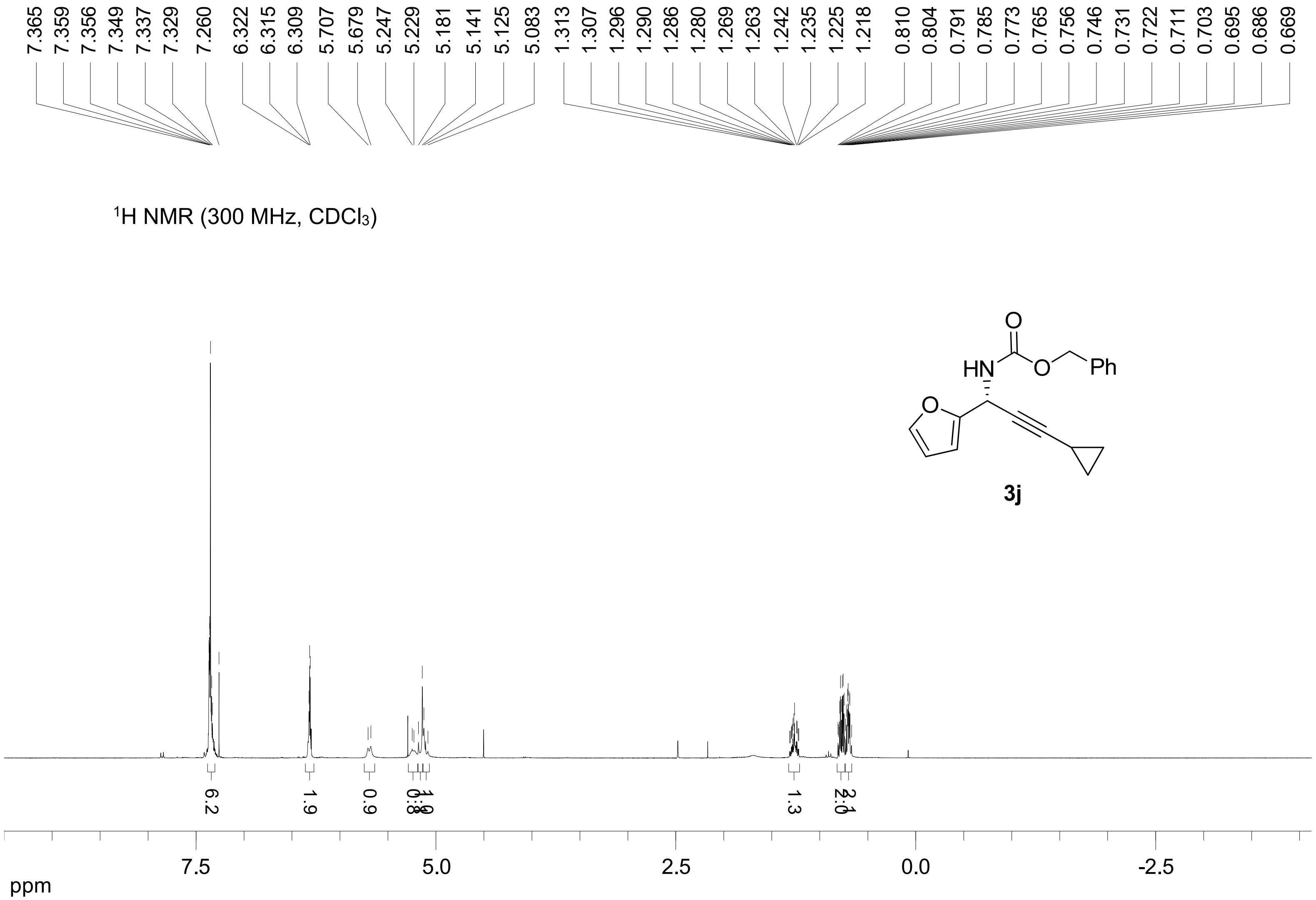

3j

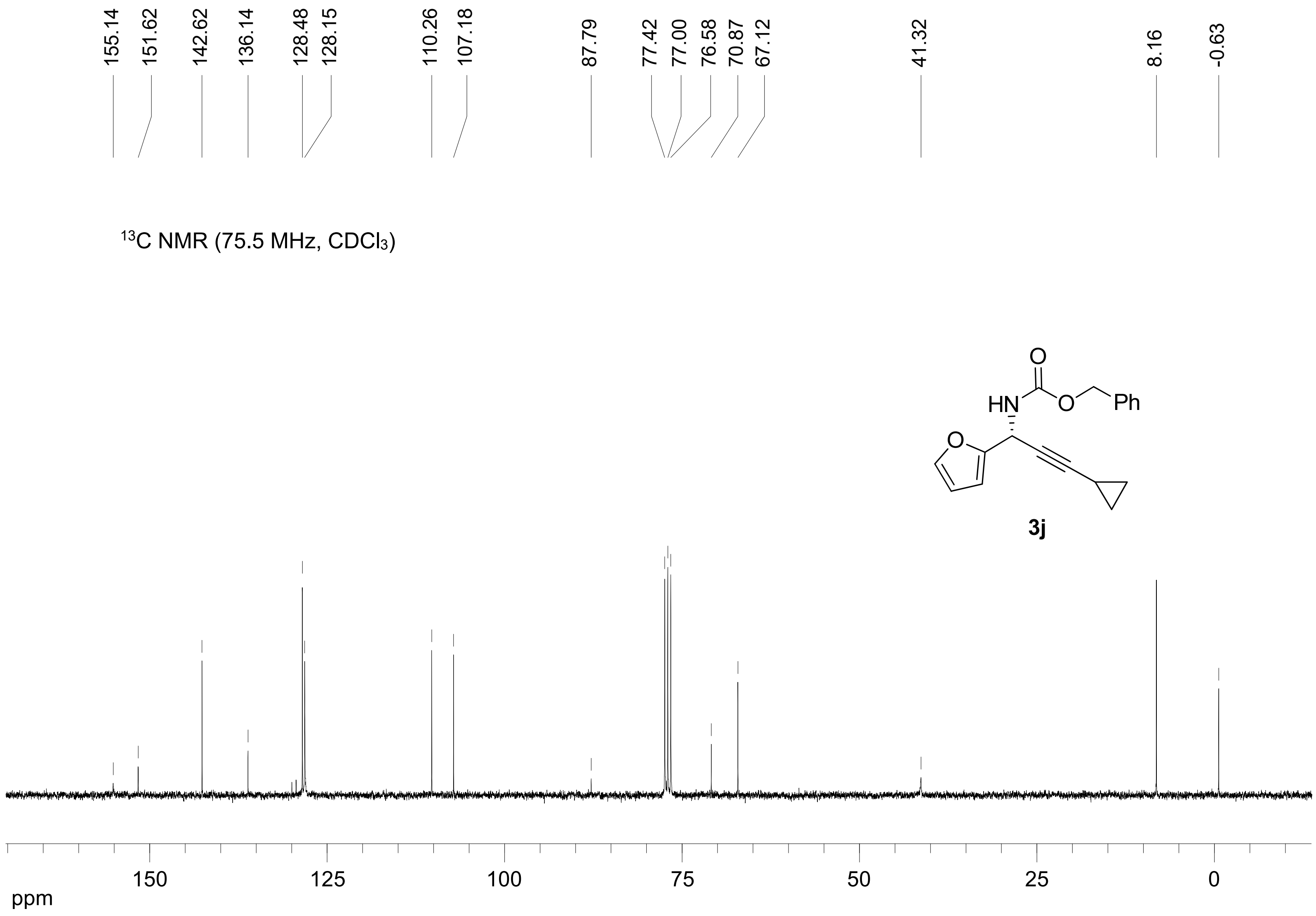
155.14
151.62
142.62
136.14
128.48
128.15
110.26
107.18
87.79
77.42
77.00
76.58
70.87
67.12
41.32
8.16
-0.63
^{13}C NMR (75.5 MHz, $CDCl_3$)
O
HN
O
Ph
O
3j
150
125
100
75
50
25
0
ppm

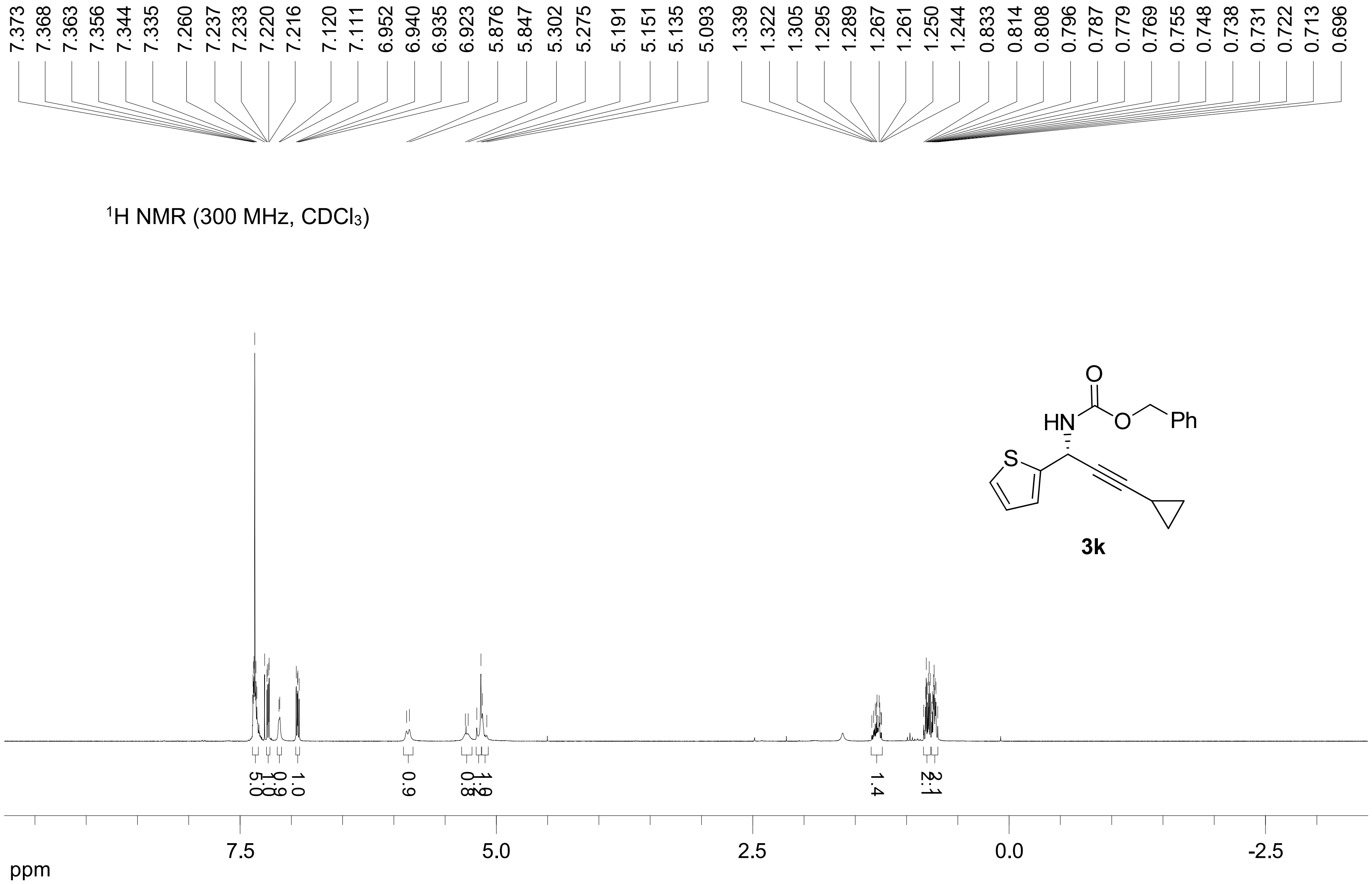
7.373 7.368 7.363 7.356 7.344 7.335 7.260 7.237 7.233 7.220 7.216 7.120 7.111 6.952 6.940 6.935 6.923 5.876 5.847 5.302 5.275 5.191 5.151 5.135 5.093 1.339 1.322 1.305 1.295 1.289 1.267 1.261 1.250 1.244 0.833 0.814 0.808 0.796 0.787 0.779 0.769 0.755 0.748 0.738 0.731 0.722 0.713 0.696
^{1}H NMR (300 MHz, $CDCl_3$)
HN
O
O
Ph
S
3k
5.0 1.0 0.9 1.0 0.9 0.8 1.0 1.4 2.1
7.5
5.0
2.5
0.0
-2.5
ppm

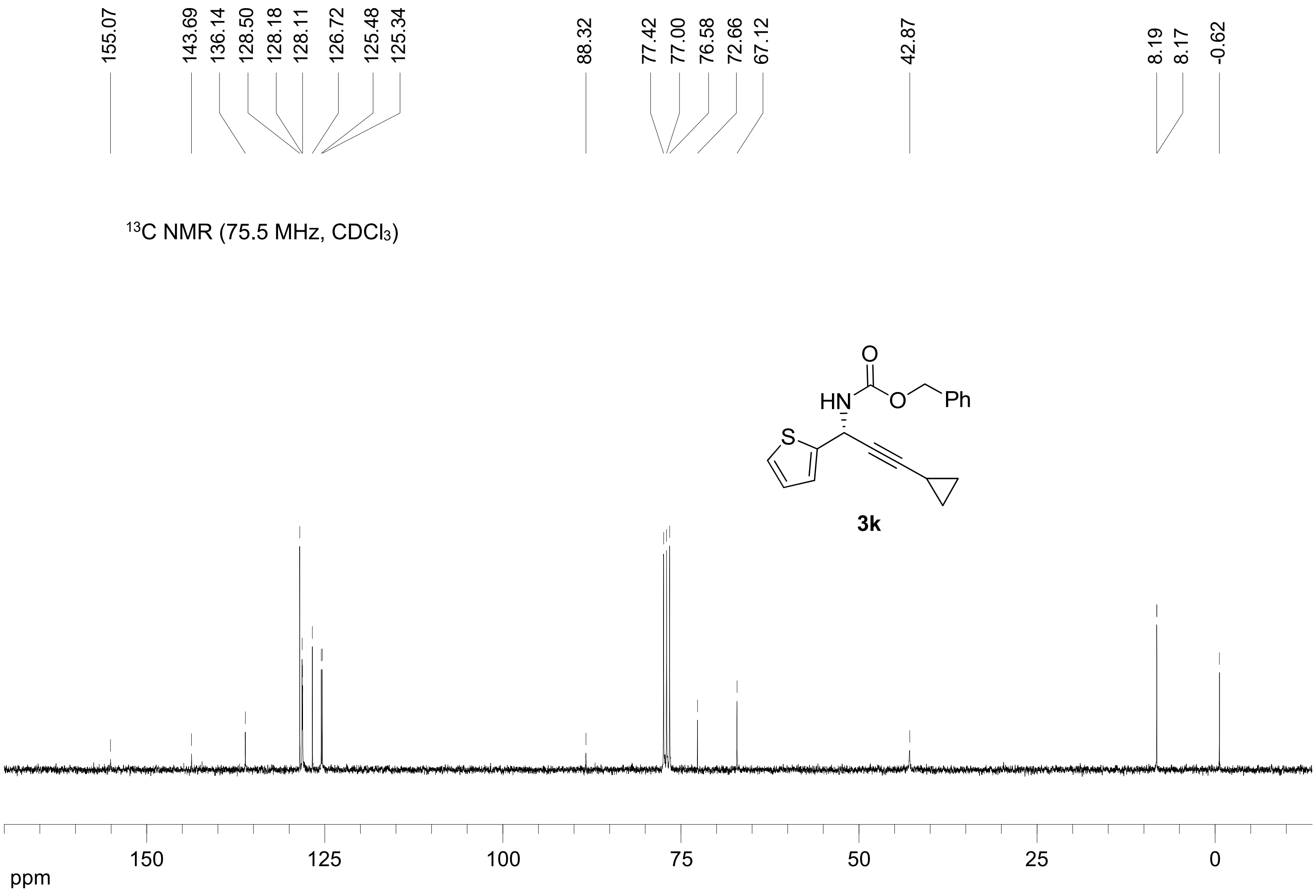
155.07
143.69
136.14
128.50
128.18
128.11
126.72
125.48
125.34
88.32
77.42
77.00
76.58
72.66
67.12
42.87
8.19
8.17
-0.62
13C NMR (75.5 MHz, CDCl3)
O
HN
O
Ph
S
3k
150
125
100
75
50
25
0
ppm

^{1}H NMR (300 MHz, $CDCl_3$)

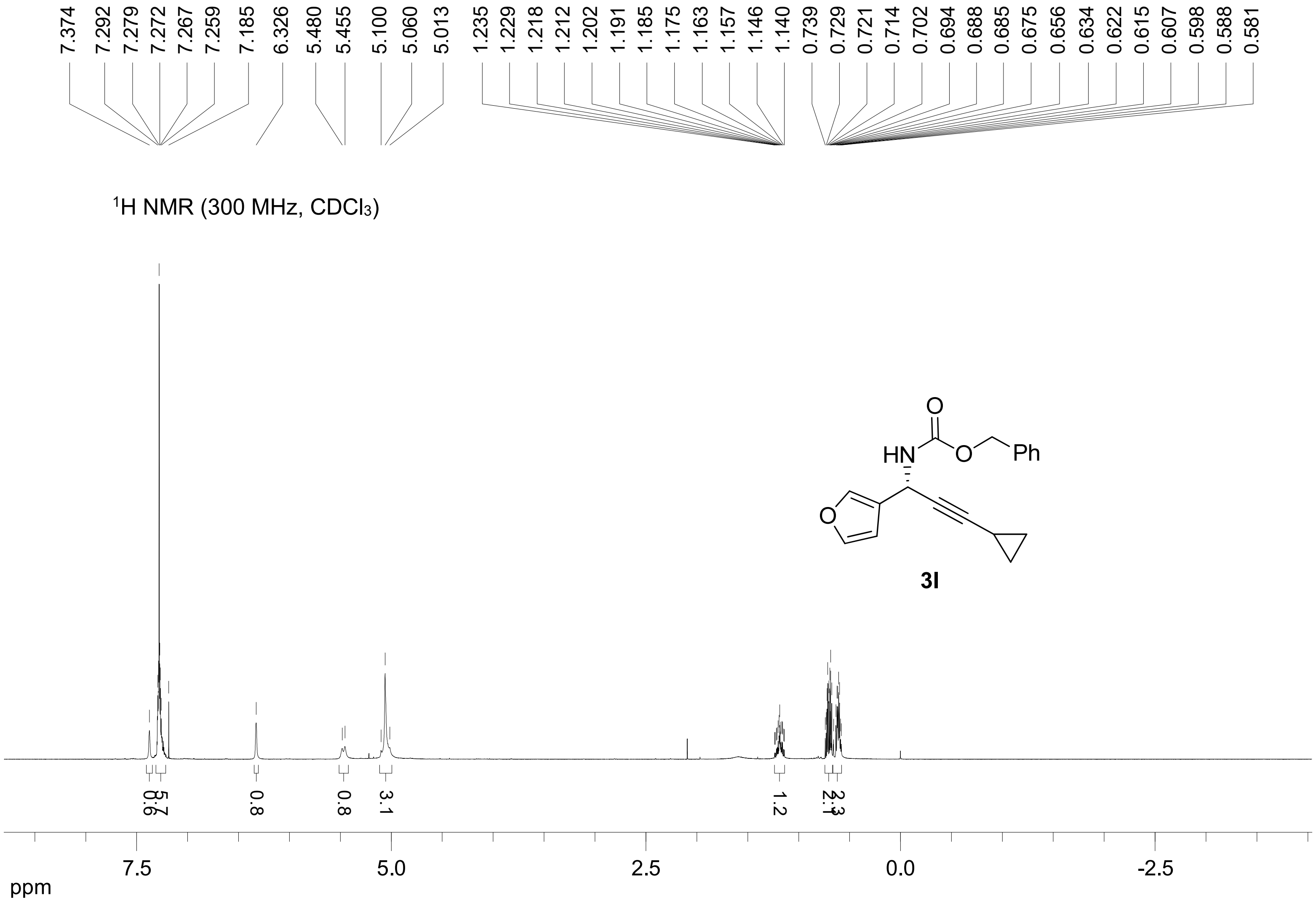

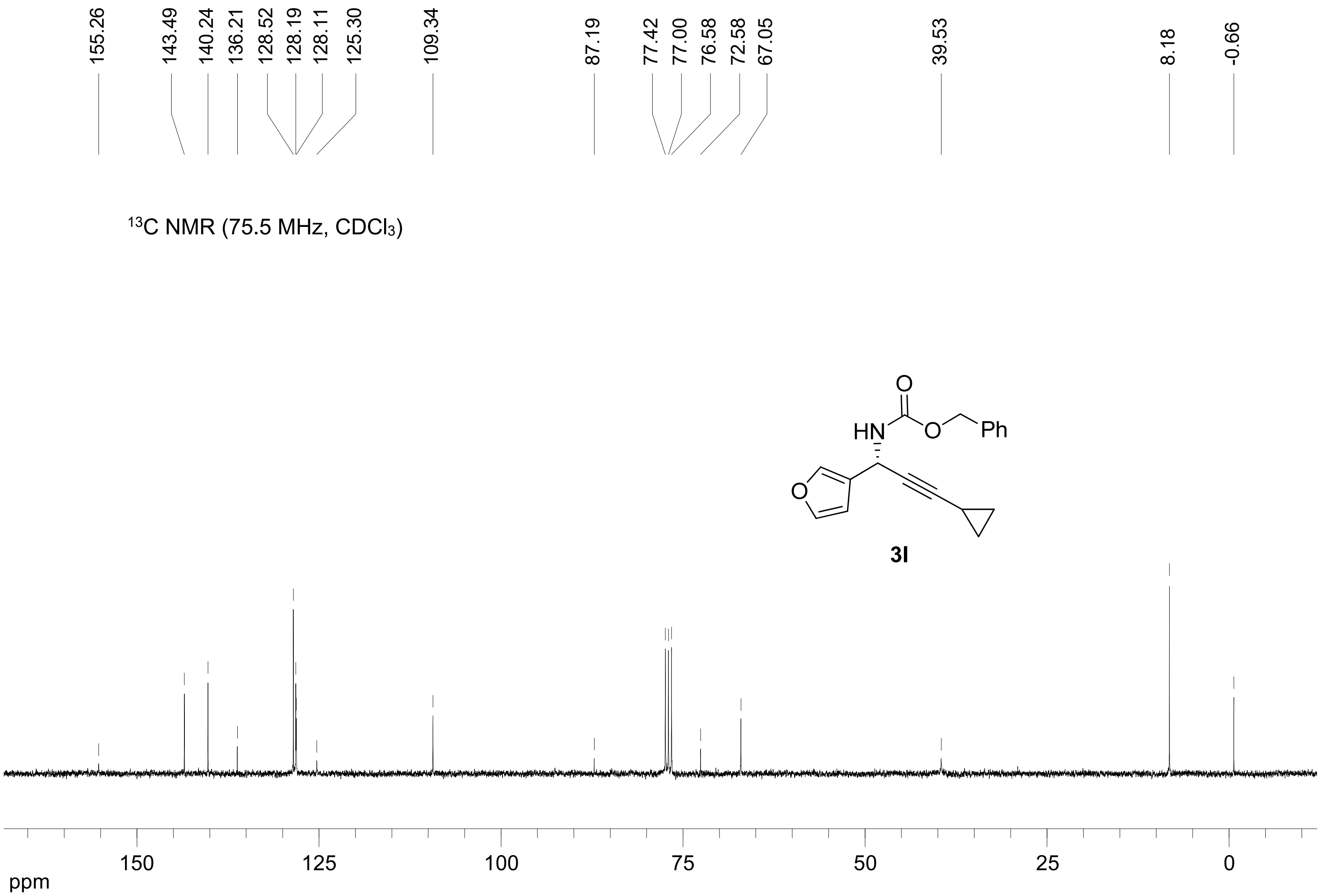
155.26
143.49
140.24
136.21
128.52
128.19
128.11
125.30
109.34
87.19
77.42
77.00
76.58
72.58
67.05
39.53
8.18
-0.66
^{13}C NMR (75.5 MHz, $CDCl_3$)
O
HN
O
Ph
O
150
125
100
75
50
25
0
ppm

3l

^1H NMR (300 MHz, $CDCl_3$)

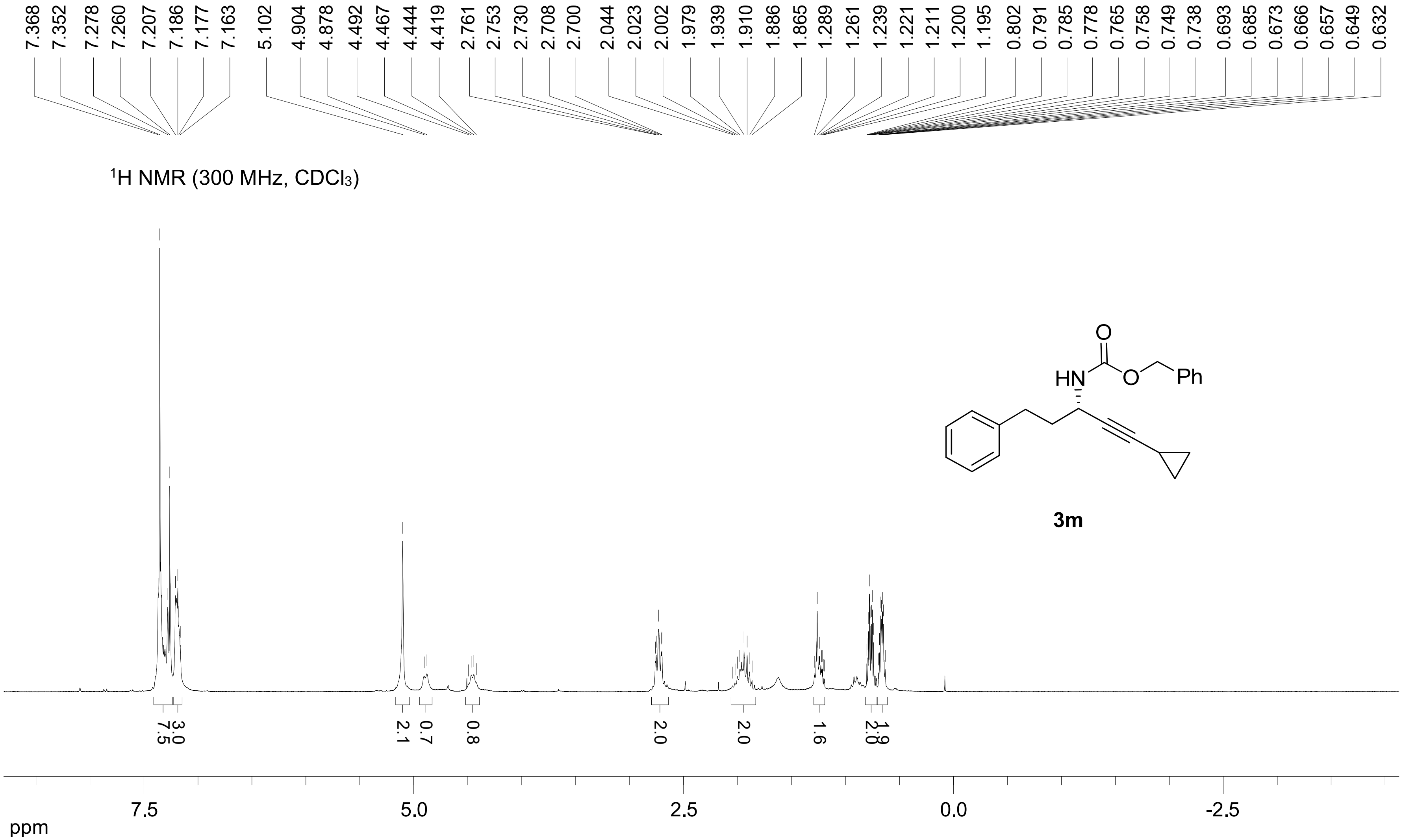

3m

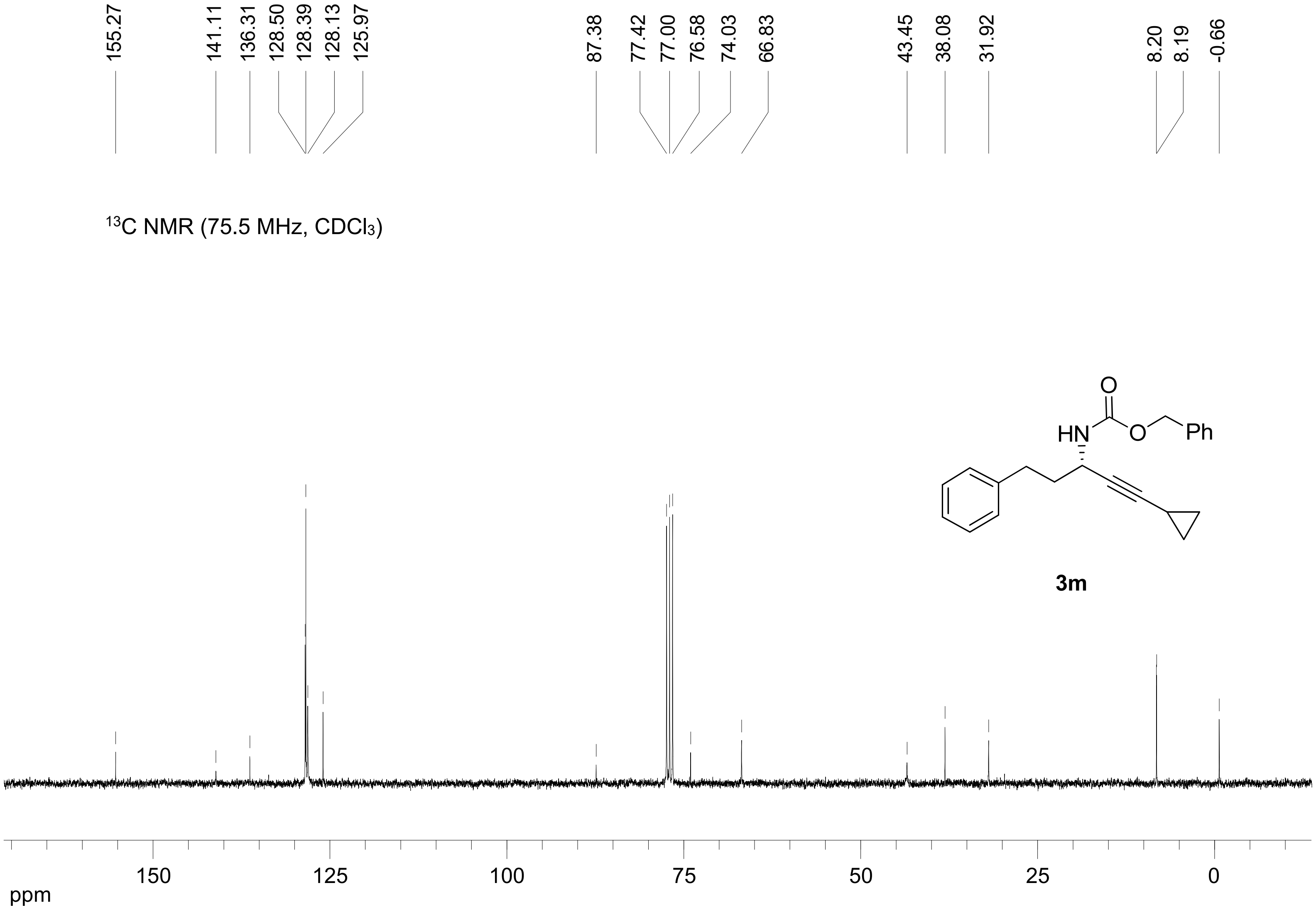

^{13}C NMR (75.5 MHz, $CDCl_3$)

3m

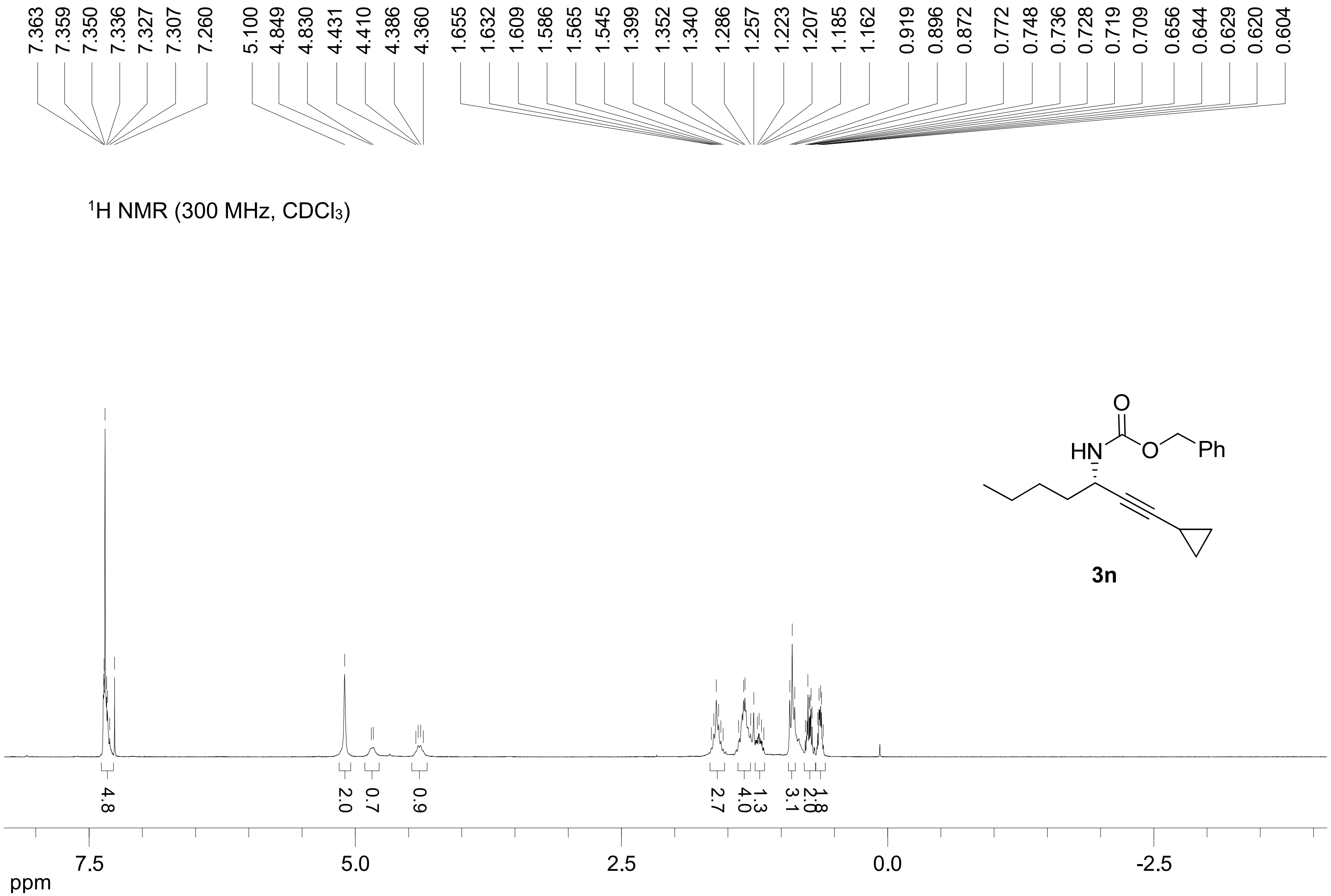
7.363 7.359 7.350 7.336 7.327 7.307 7.260
5.100 4.849 4.830 4.431 4.410 4.386 4.360
1.655 1.632 1.609 1.586 1.565 1.545 1.399 1.352 1.340 1.286 1.257 1.223 1.207 1.185 1.162
0.919 0.896 0.872 0.772 0.748 0.736 0.728 0.719 0.709 0.656 0.644 0.629 0.620 0.604
^{1}H NMR (300 MHz, $CDCl_3$)
HN
O
O
Ph
3n
4.8 2.0 0.7 0.9 2.7 4.0 1.3 3.1 2.0 1.8
7.5
5.0
2.5
0.0
-2.5
ppm

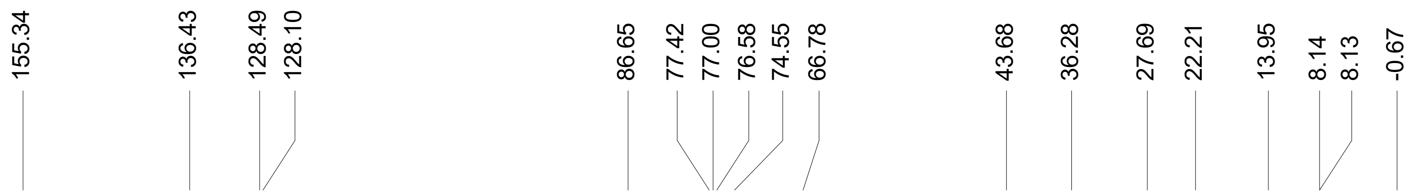

^{13}C NMR (75.5 MHz, $CDCl_3$)

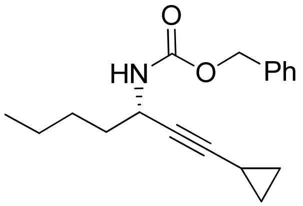

3n

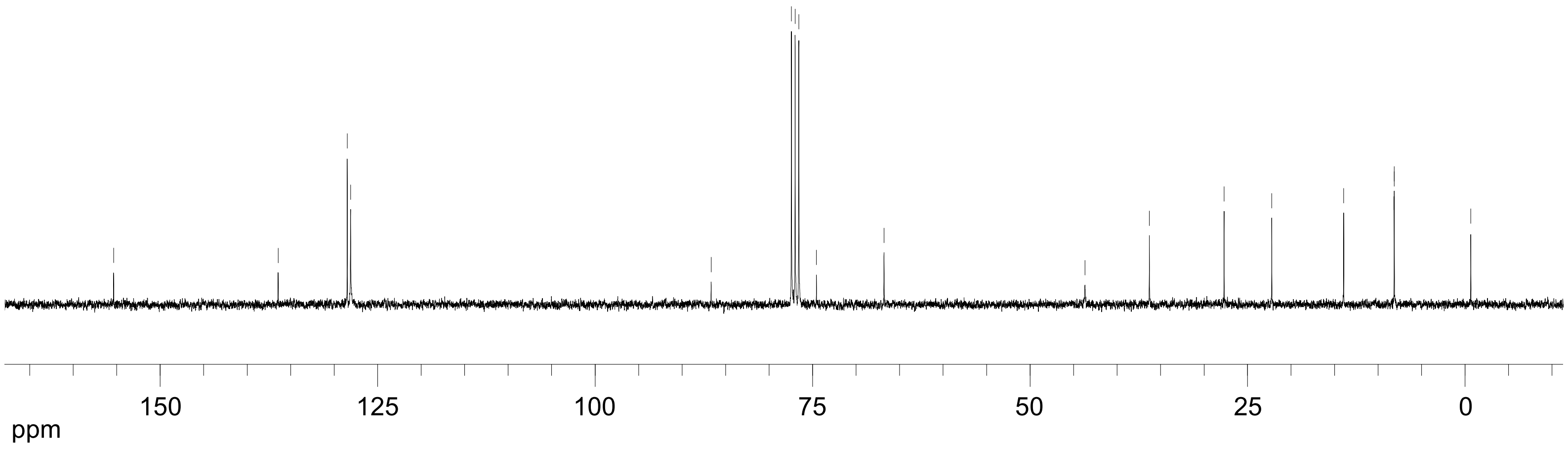

^{1}H NMR (300 MHz, $CDCl_3$)

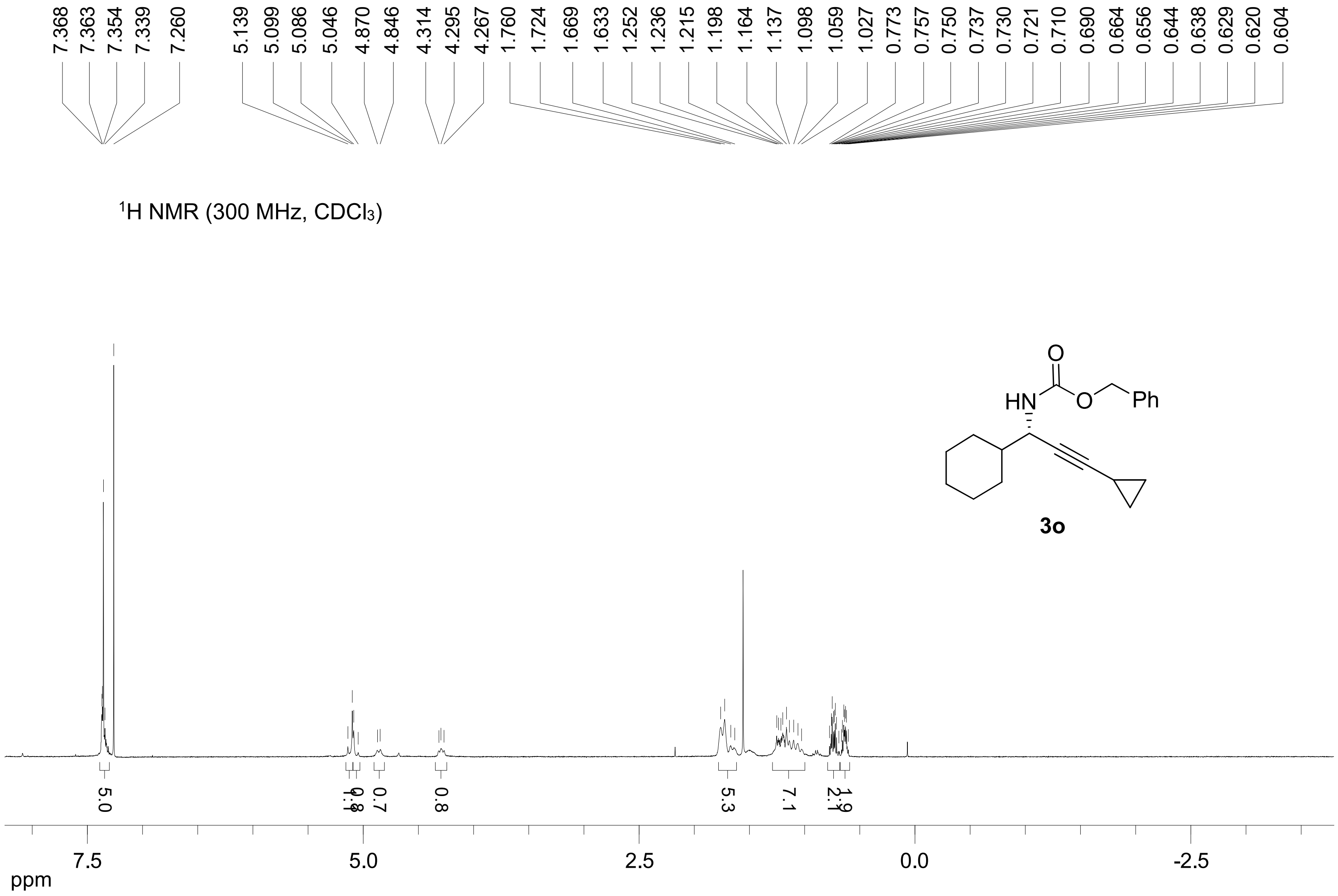

3o

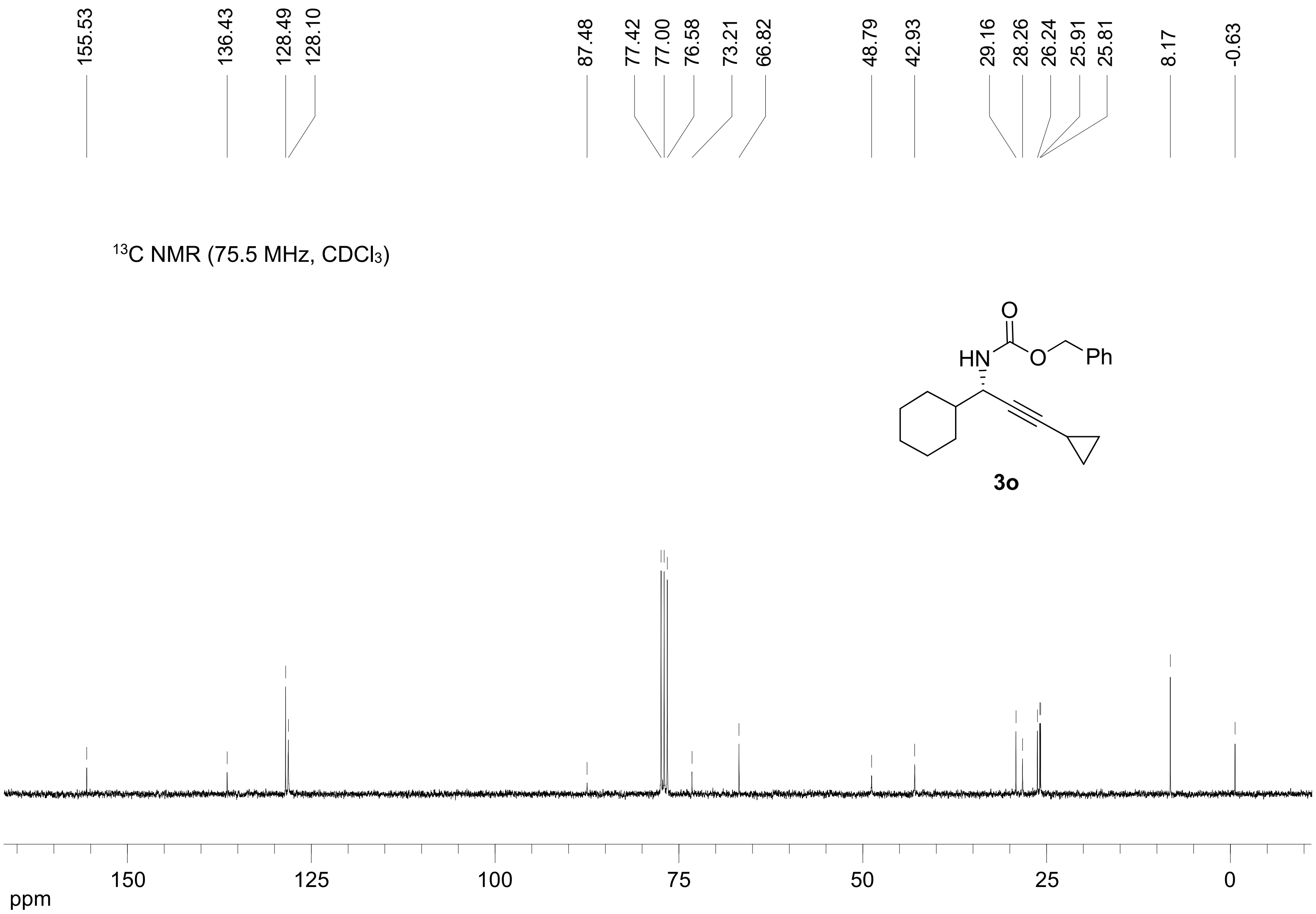
155.53
136.43
128.49
128.10
87.48
77.42
77.00
76.58
73.21
66.82
48.79
42.93
29.16
28.26
26.24
25.91
25.81
8.17
-0.63
^{13}C NMR (75.5 MHz, $CDCl_3$)
O
HN
O
Ph
150
125
100
75
50
25
0
ppm

3o

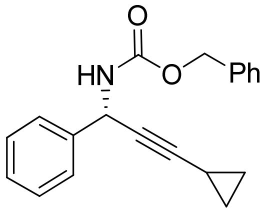

3a

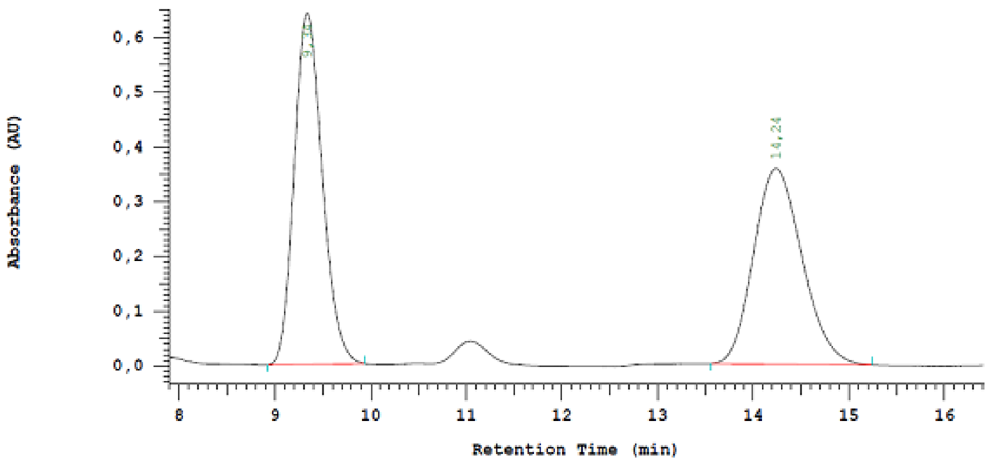

No.	RT	Area	Area %
1	9,34	6556915	51,056
2	14,24	6285755	48,944
		12842670	100,000

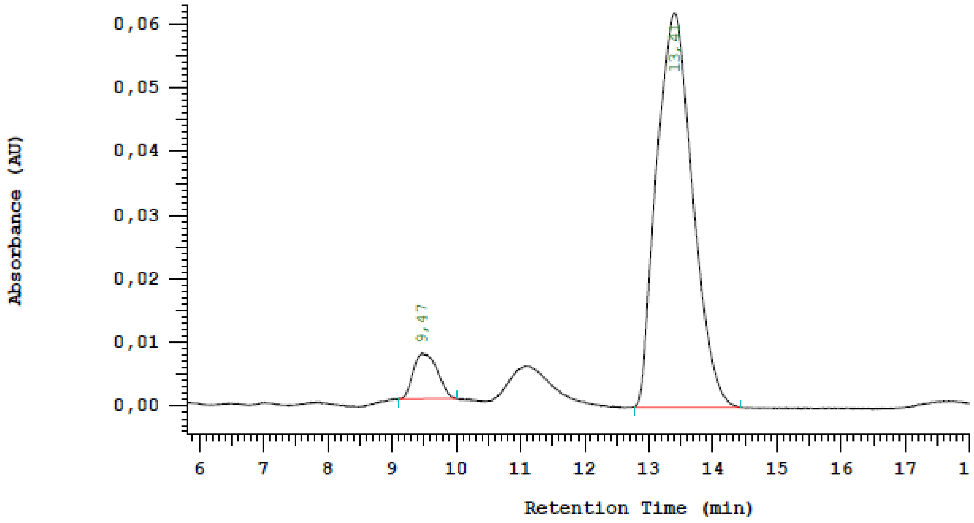

No.	RT	Area	Area %
1	9,47	92110	7,083
2	13,41	1208280	92,917
		1300390	100,000

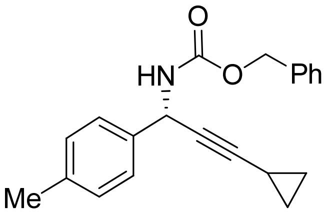

3b

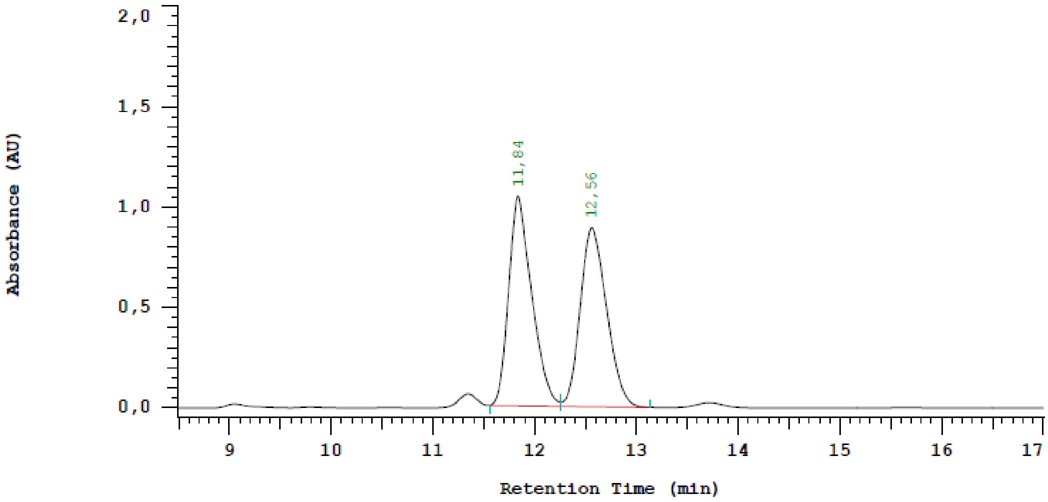

No.	RT	Area	Area %
1	11,84	8240919	50,669
2	12,56	8023147	49,331
		16264066	100,000

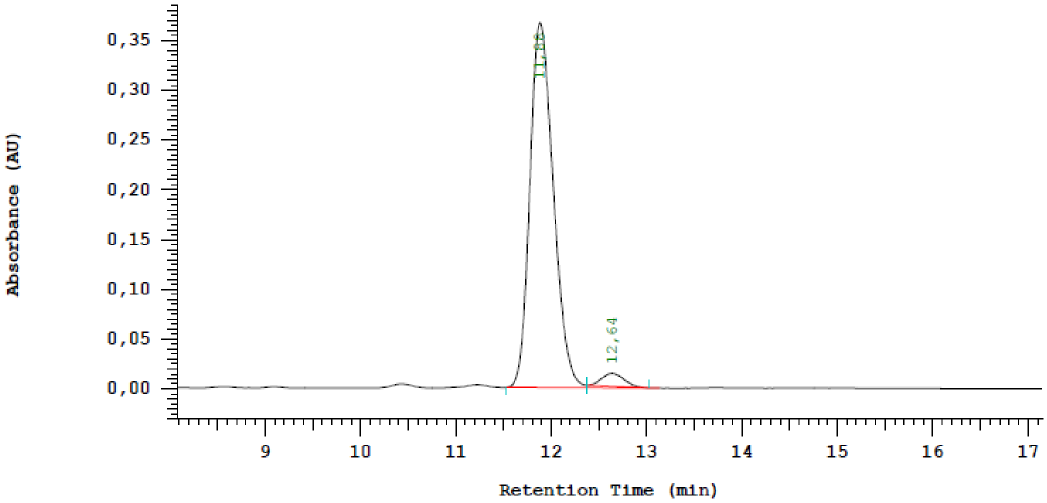

No.	RT	Area	Area %
1	11,88	3156903	96,713
2	12,64	107300	3,287
		3264203	100,000

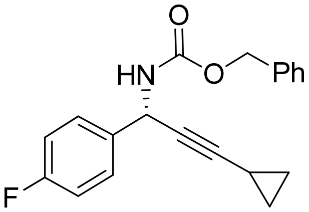

3c

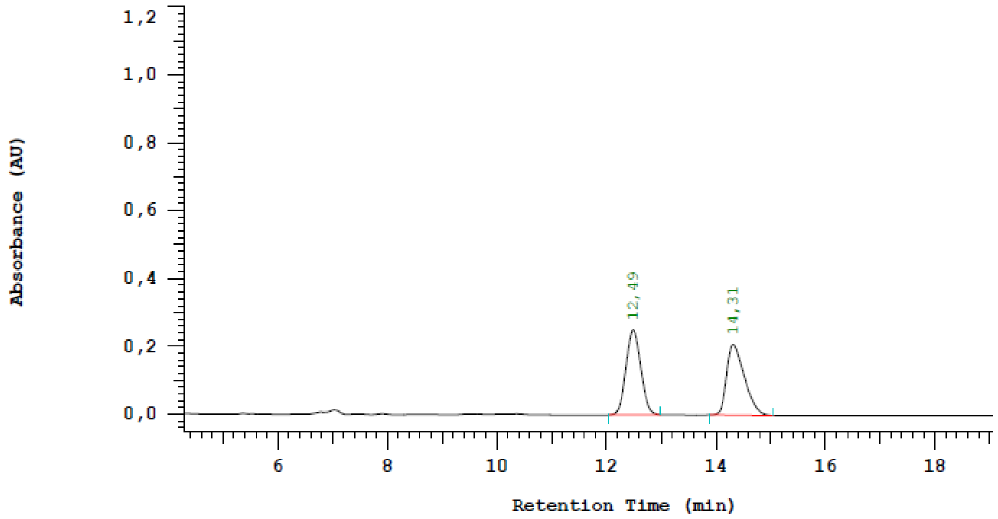

No.	RT	Area	Area %
1	12,49	2360151	50,001
2	14,31	2360036	49,999
		4720187	100,000

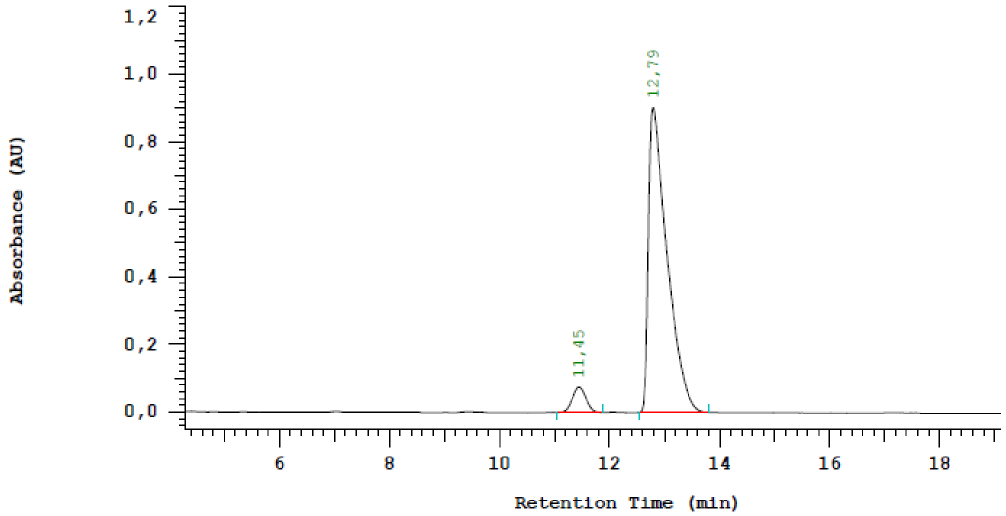

No.	RT	Area	Area %
1	11,45	645844	5,750
2	12,79	10585616	94,250
		11231460	100,000

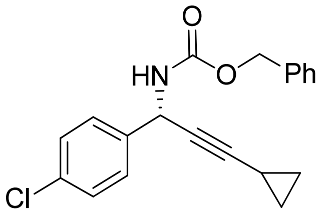

3d

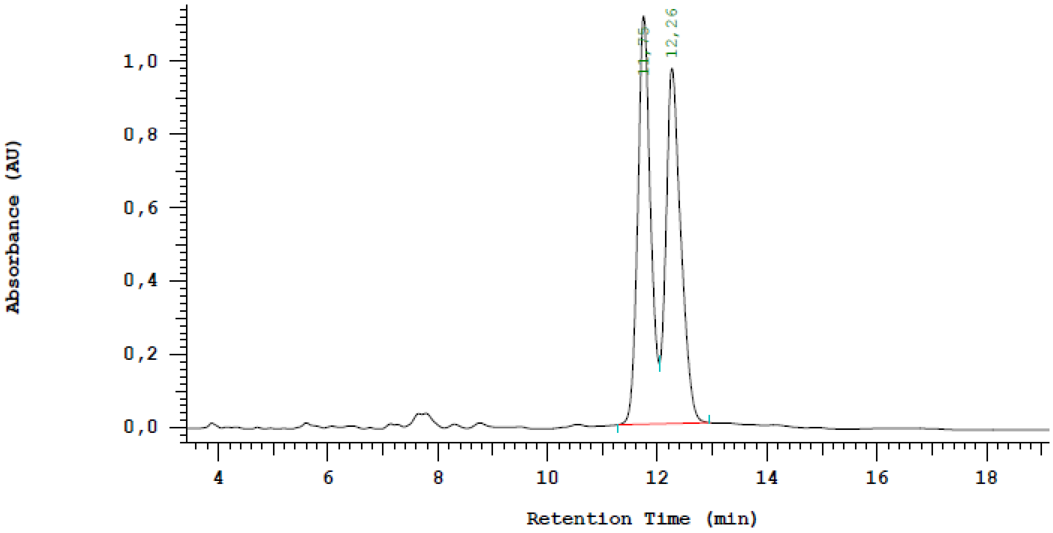

No.	RT	Area	Area %
1	11,75	9052621	49,759
2	12,26	9140204	50,241
		18192825	100,000

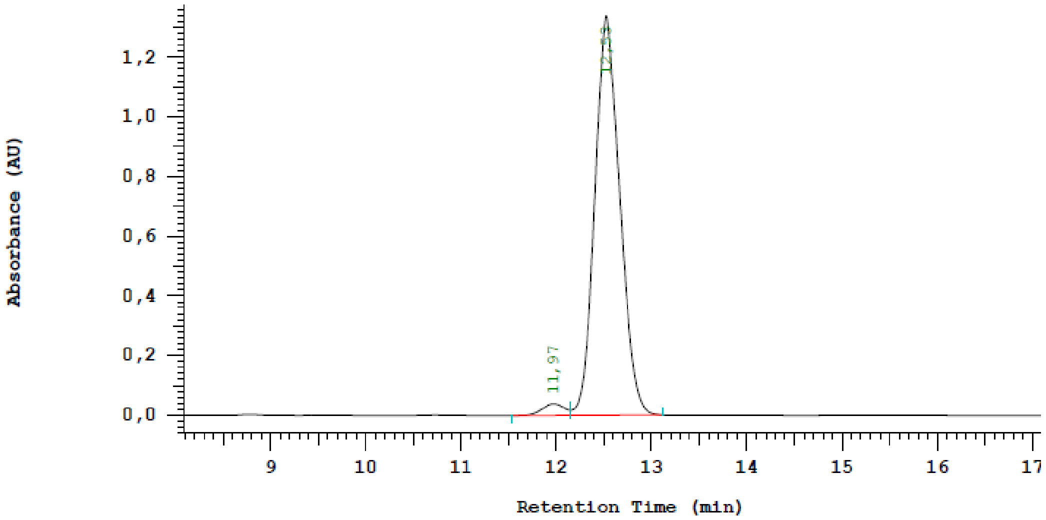

No.	RT	Area	Area %
1	11,97	315448	2,529
2	12,53	12158253	97,471
		12473701	100,000

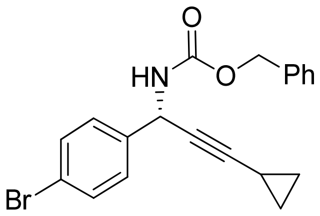

3e

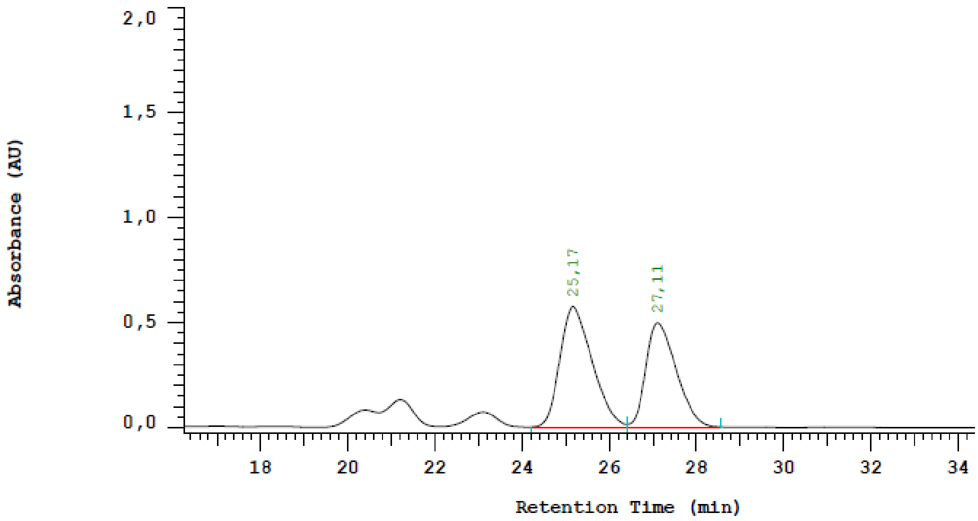

No.	RT	Area	Area %
1	25,17	14742550	55,092
2	27,11	12017348	44,908
		26759898	100,000

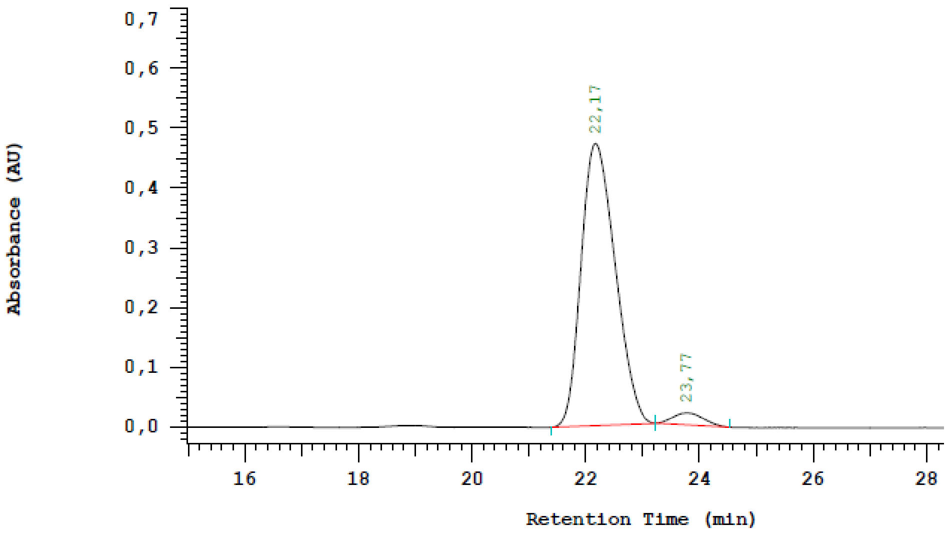

No.	RT	Area	Area %
1	22,17	9822529	96,196
2	23,77	388460	3,804
		10210989	100,000

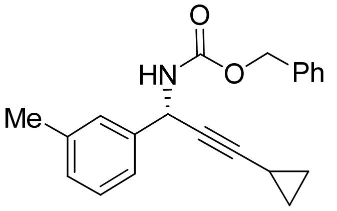

3f

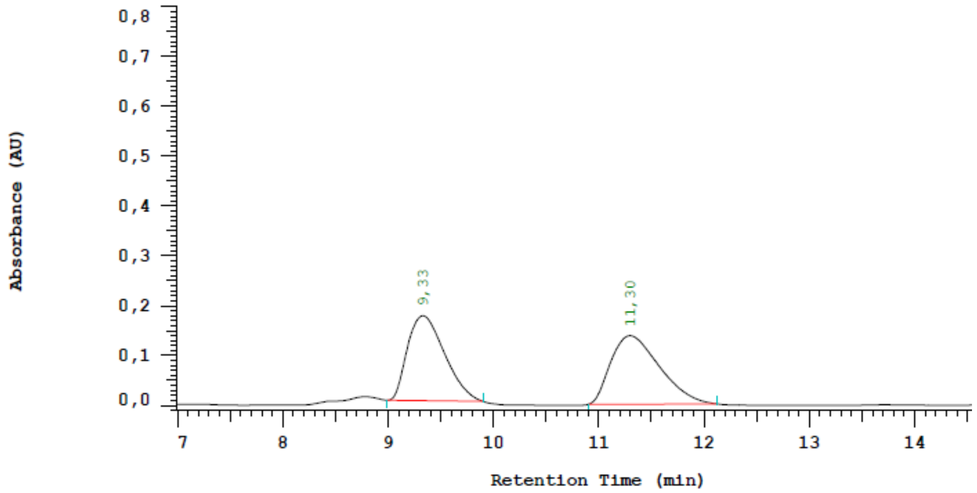

No.	RT	Area	Area %
1	9,33	2094836	48,579
2	11,30	2217426	51,421
		4312262	100,000

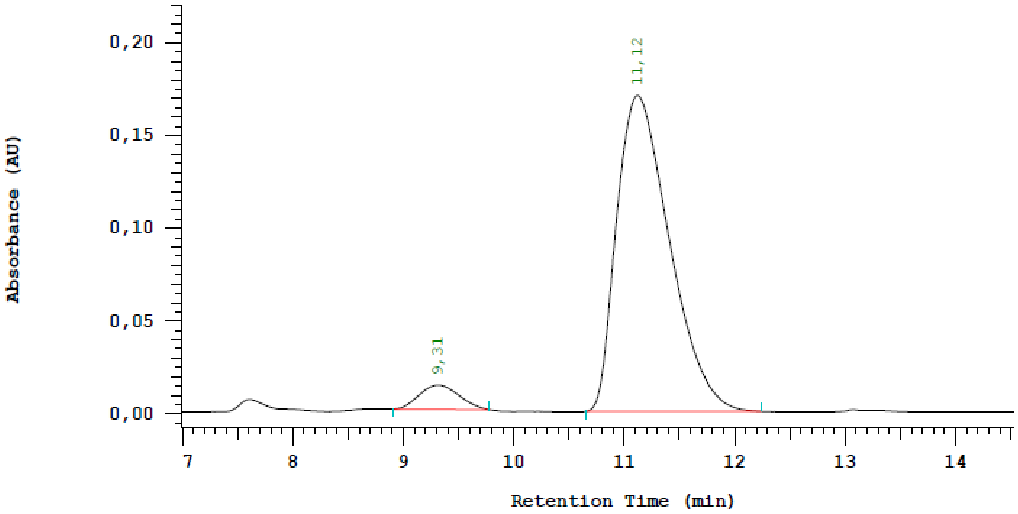

No.	RT	Area	Area %
1	9,31	162658	5,435
2	11,12	2830006	94,565
		2992664	100,000

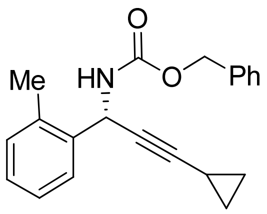

3g

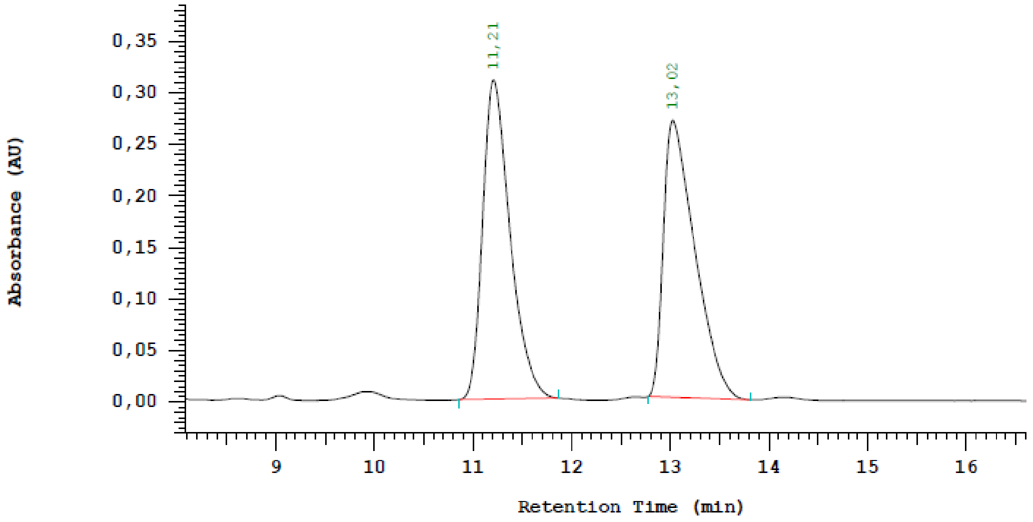

No.	RT	Area	Area %
1	11,21	2993501	50,107
2	13,02	2980752	49,893
		5974253	100,000

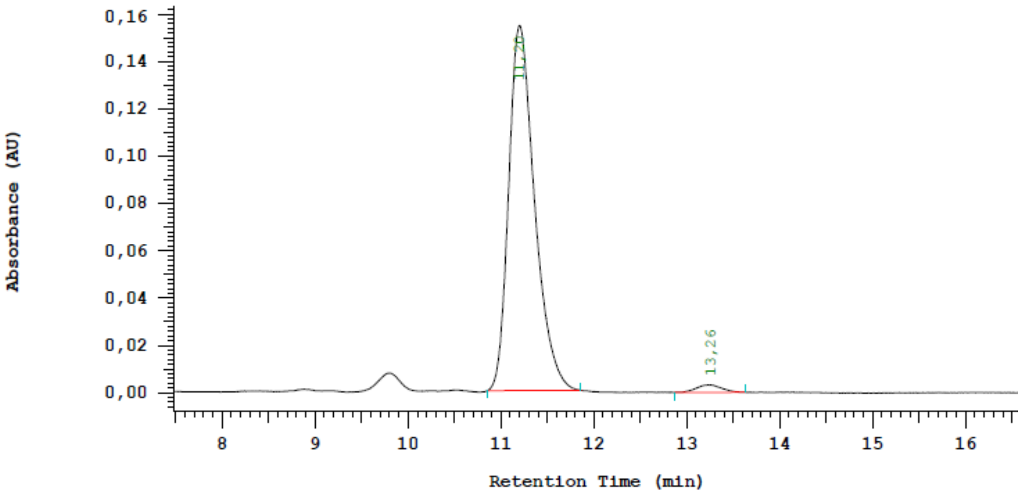

No.	RT	Area	Area %
1	11,20	1493130	98,079
2	13,26	29240	1,921
		1522370	100,000

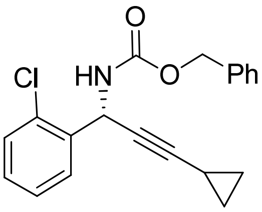

3h

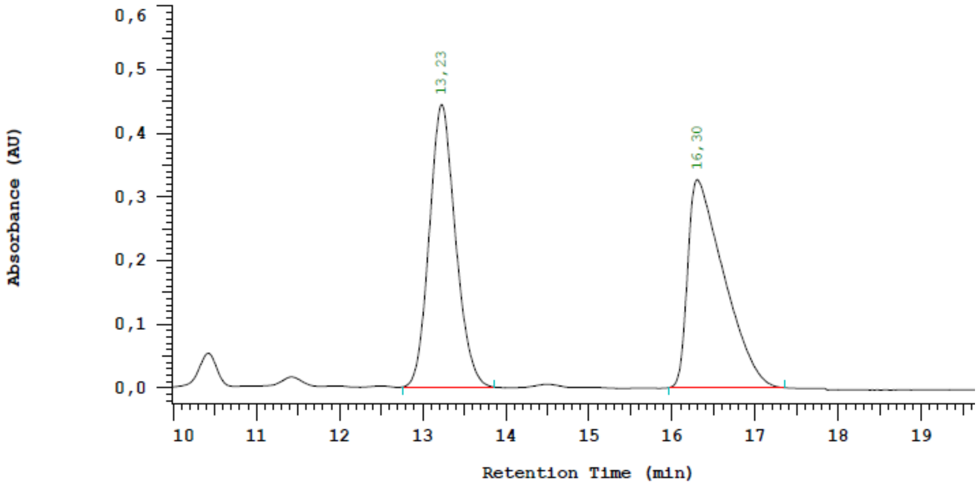

No.	RT	Area	Area %
1	13,23	4857082	49,542
2	16,30	4946947	50,458
		9804029	100,000

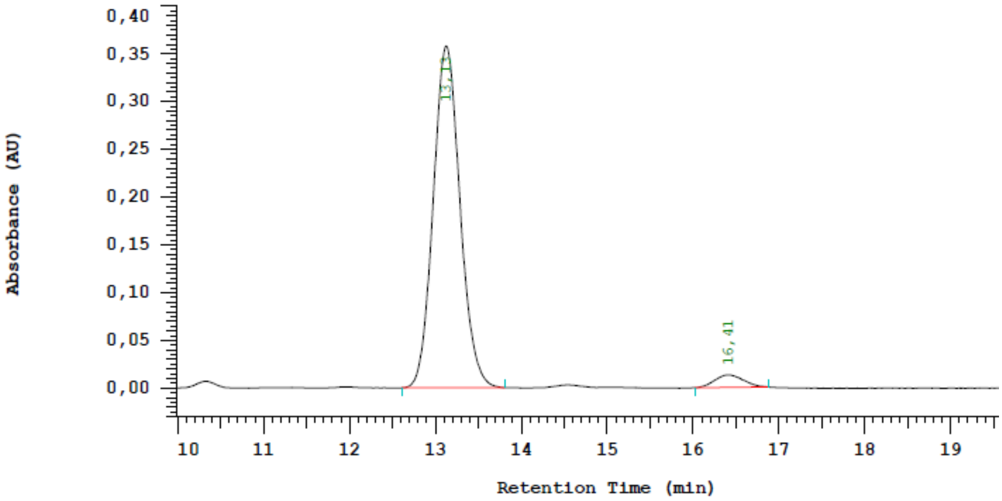

No.	RT	Area	Area %
1	13,13	3870819	96,215
2	16,41	152273	3,785
		4023092	100,000

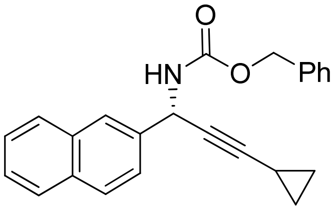

3i

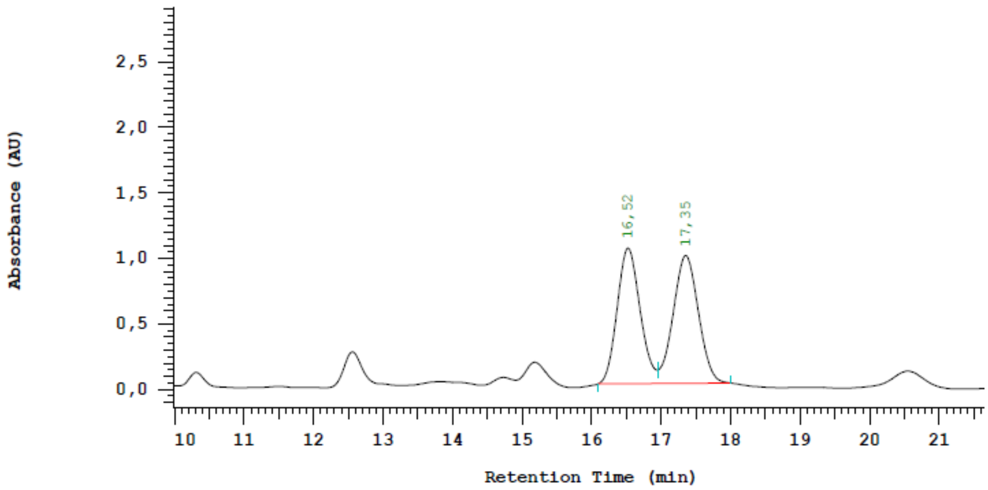

No.	RT	Area	Area %
1	16,52	11858385	48,964
2	17,35	12360367	51,036
		24218752	100,000

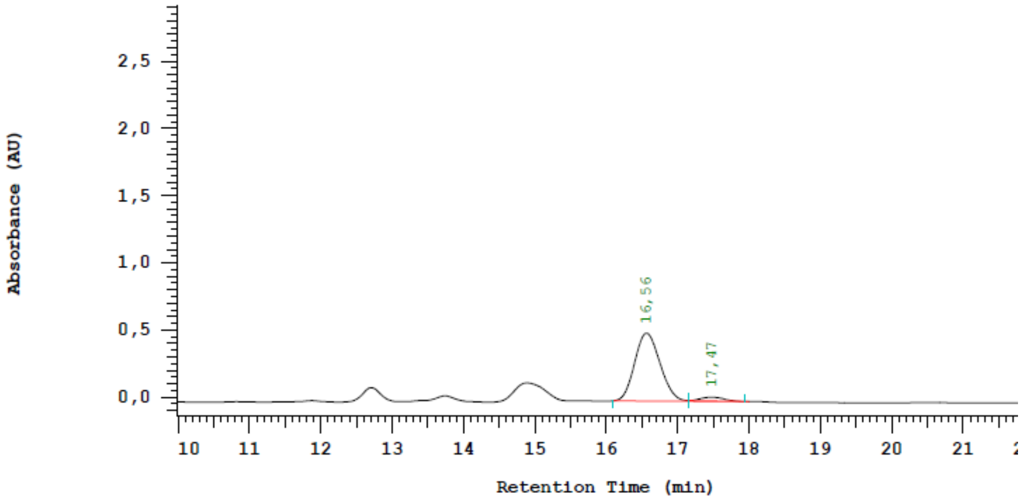

No.	RT	Area	Area %
1	16,56	6295235	95,246
2	17,47	314215	4,754
		6609450	100,000

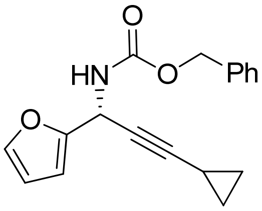

3j

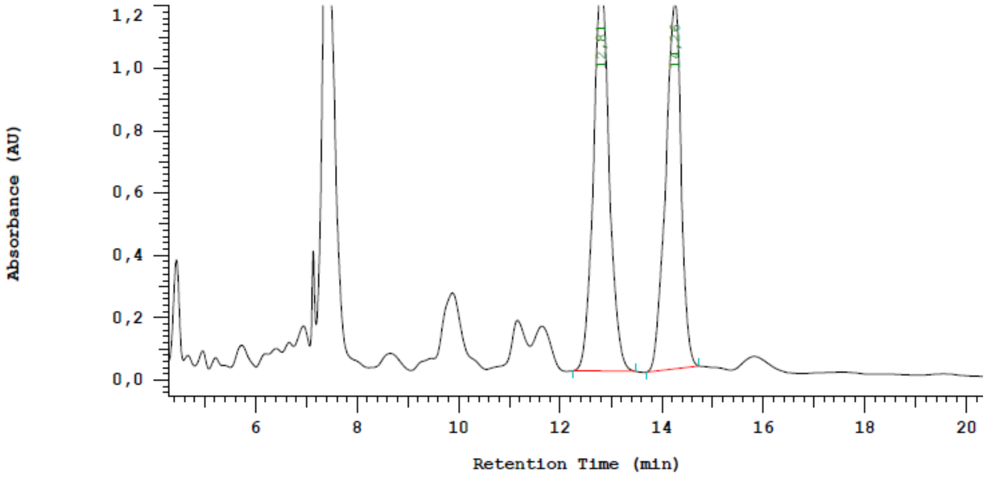

No.	RT	Area	Area %
1	12,81	13563224	52,004
2	14,26	12517715	47,996
		26080939	100,000

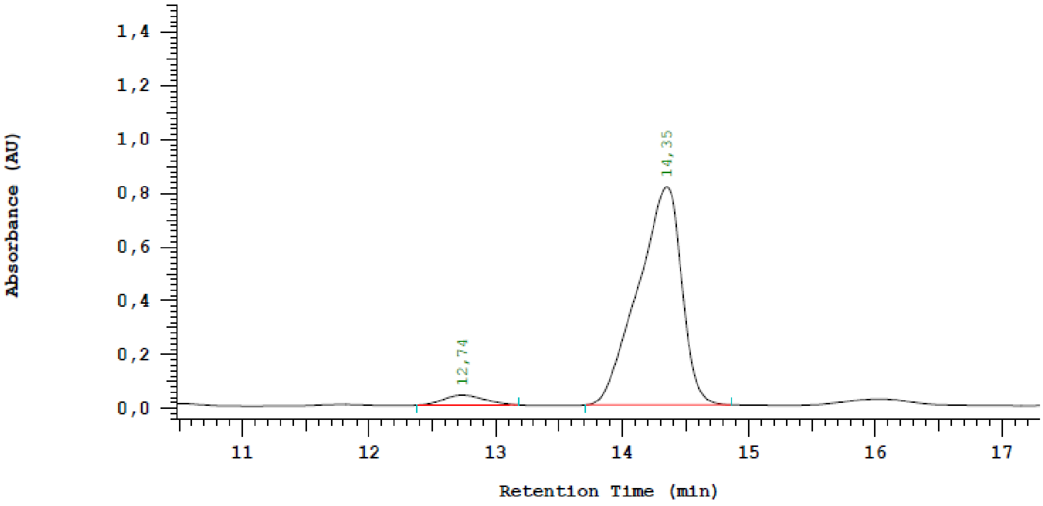

No.	RT	Area	Area %
1	12,74	418610	4,126
2	14,35	9727871	95,874
		10146481	100,000

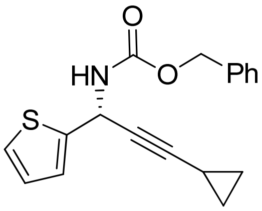

3k

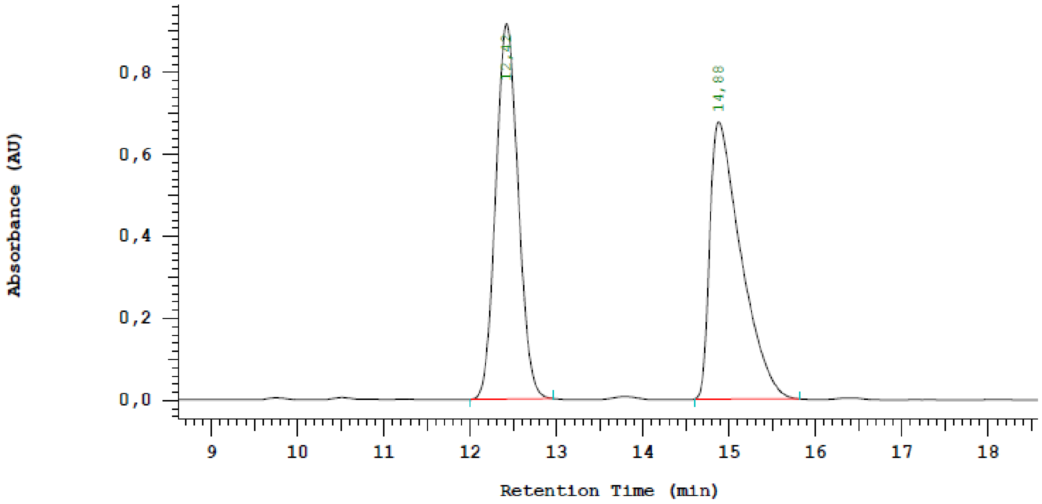

No.	RT	Area	Area %
1	12,42	8271110	49,437
2	14,88	8459483	50,563
		16730593	100,000

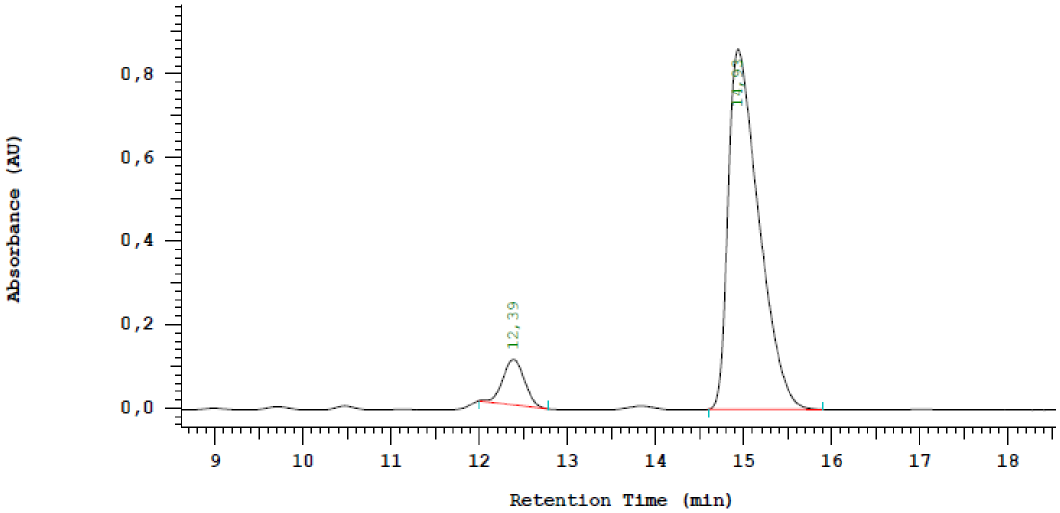

No.	RT	Area	Area %
1	12,39	931576	8,225
2	14,93	10393976	91,775
		11325552	100,000

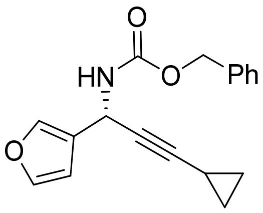

3l

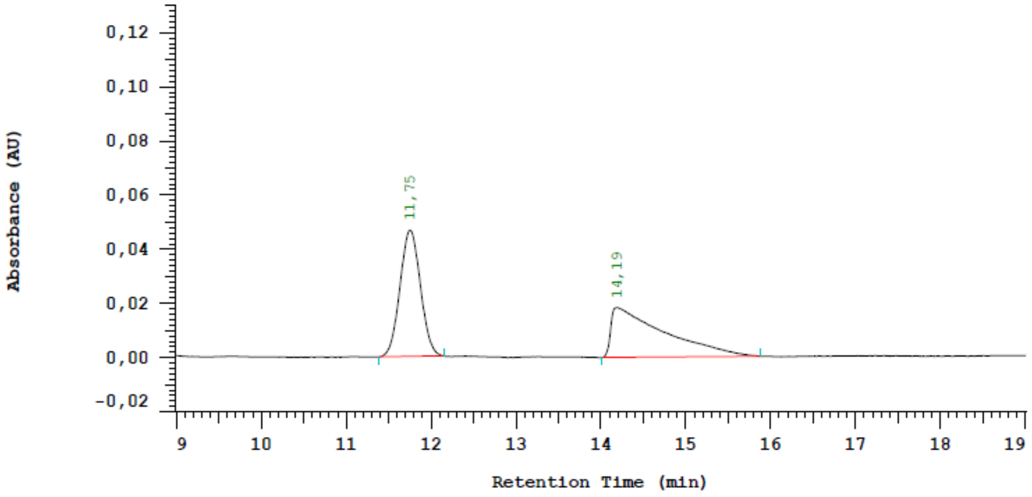

No.	RT	Area	Area %
1	11,75	391252	49,410
2	14,19	400601	50,590
		791853	100,000

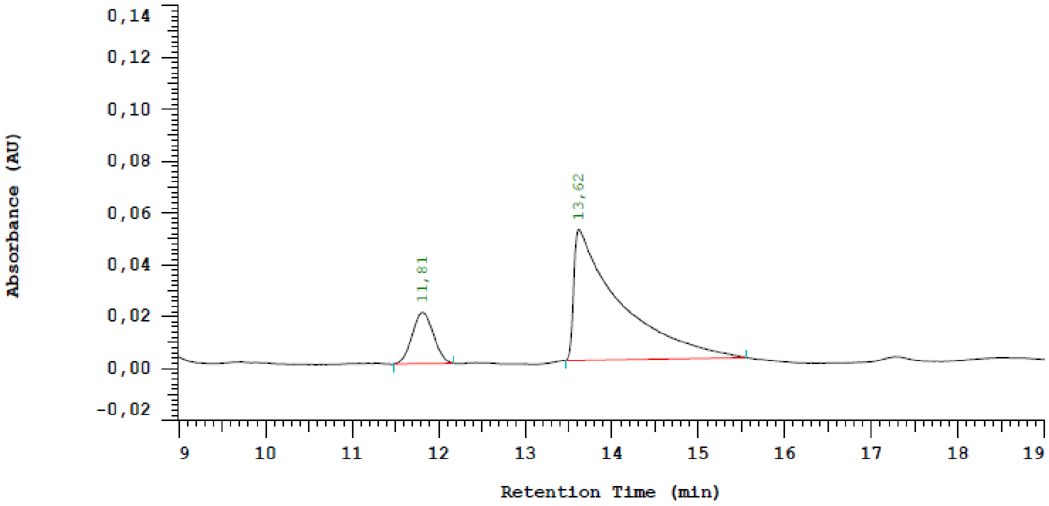

No.	RT	Area	Area %
1	11,81	168789	15,329
2	13,62	932342	84,671
		1101131	100,000

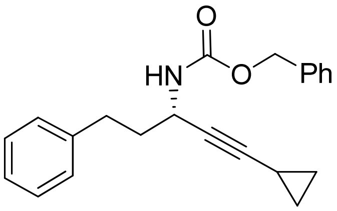

3m

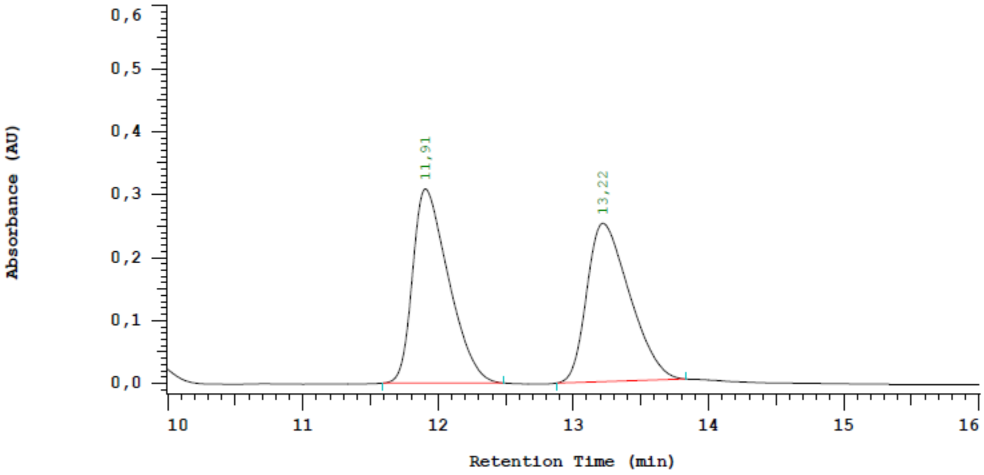

No.	RT	Area	Area %
1	11,91	2848510	50,831
2	13,22	2755335	49,169
		5603845	100,000

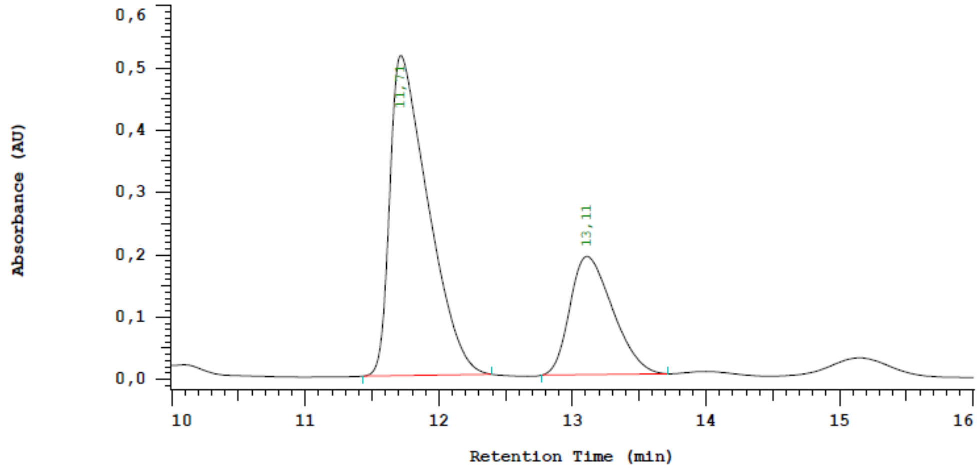

No.	RT	Area	Area %
1	11,71	5110850	70,799
2	13,11	2107921	29,201
		7218771	100,000

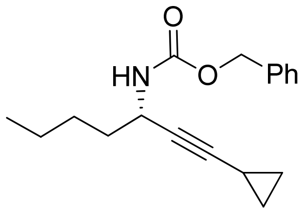

3n

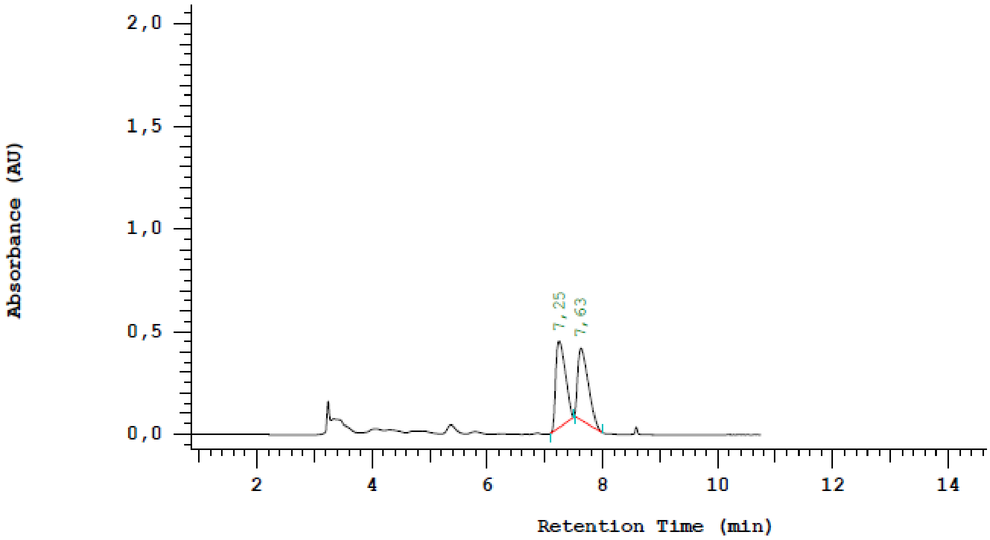

No.	RT	Area	Area %
1	7,25	2340221	50,950
2	7,63	2252979	49,050
		4593200	100,000

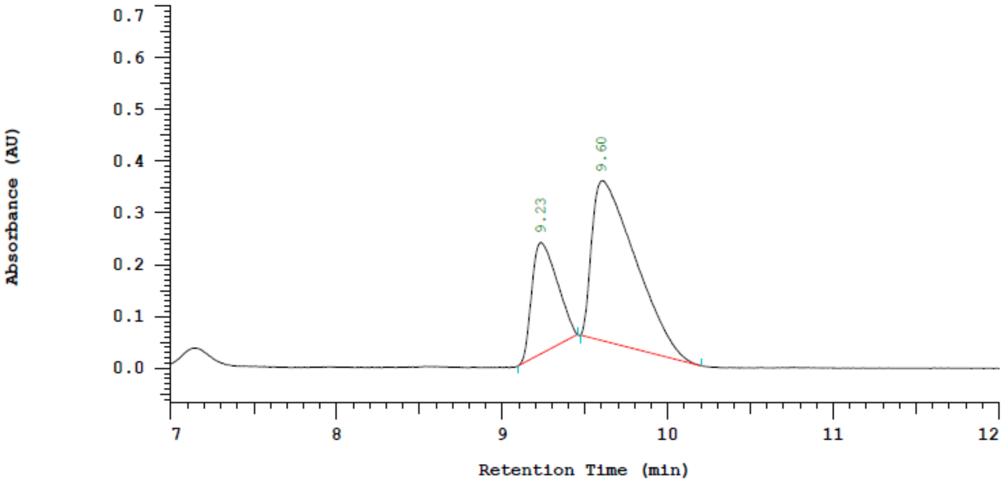

No.	RT	Area	Area %
1	9.23	1126880	28.325
2	9.60	2851470	71.675
		3978350	100.000

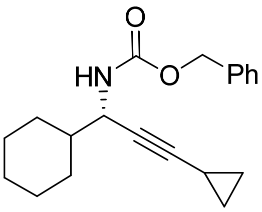

3o

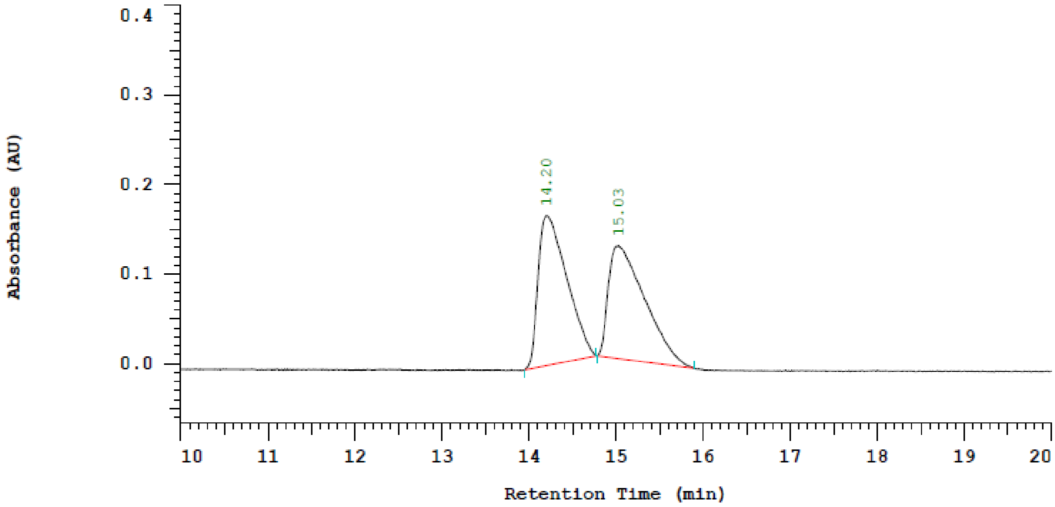

No.	RT	Area	Area %
1	14.20	1902340	50.768
2	15.03	1844810	49.232
		3747150	100.000

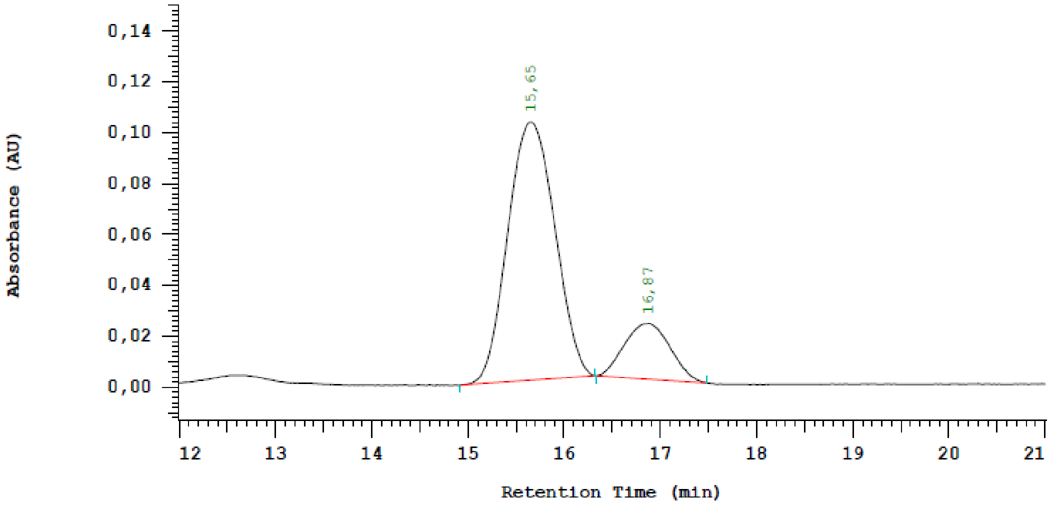

No.	RT	Area	Area %
1	15,65	1722941	82,636
2	16,87	362045	17,364
		2084986	100,000
